# Shared and unique genomic structural variants of different histological components within testicular germ cell tumours identified with mate pair sequencing

**DOI:** 10.1038/s41598-019-39956-y

**Published:** 2019-03-05

**Authors:** Alan H. Bryce, Jan B. Egan, James B. Smadbeck, Sarah H. Johnson, Stephen J. Murphy, Faye R. Harris, Geoffrey C. Halling, Simone B. S. P. Terra, John Cheville, Lance Pagliaro, Brad Leibovich, Brian A. Costello, George Vasmatzis

**Affiliations:** 1 Division of Hematology and Medical Oncology, Mayo Clinic, Phoenix, Arizona USA; 2 Mayo Clinic Cancer Center, Phoenix, Arizona USA; 3 Center for Individualized Medicine, Mayo Clinic, Rochester, Minnesota USA; 4 Department of Laboratory Medicine and Pathology, Mayo Clinic, Rochester, Minnesota USA; 5 Division of Medical Oncology, Mayo Clinic, Rochester, Minnesota USA; 6 Department of Urology, Mayo Clinic, Rochester, Minnesota USA; 7 Department of Molecular Medicine, Mayo Clinic, Rochester, Minnesota USA

## Abstract

Post-pubertal testicular germ-cell tumours (TGCTs) can present with a variety of distinct histologies which are nevertheless lineage related and often co-occurring. The exact lineage relationships and developmental pathways leading to the different histologies is debated. In order to investigate the relationship of histologic populations, mate-pair sequencing (MPseq) and exome sequencing (ExomeSeq) were conducted on different histological populations within the same tumour. Ten TGCTs with 1–3 histologic types/tumour were sequenced. Junctions of somatic chromosomal rearrangements were identified on a per genome basis, with germ cell neoplasia *in situ* possessing the least (median 1, range 0–4) and embryonal carcinoma the most (median 8.5, range 6–12). Copy number variation revealed gains and losses, including isoform 12p (i12p) (10/10 samples), and chromosomes 7, 8, and 21 gains (7/10 samples). Mapping of shared junctions within a tumour revealed lineage relationships, but only i12p was shared between patients. ExomeSeq from two cases demonstrated a high level of copy-neutral loss of heterozygosity. Parallel assessment of separate histologies within a single TGCT demonstrated cumulative and divergent changes, suggesting the importance of parallel sequencing for detection of relevant biomarkers.

## Introduction

Post-pubertal testicular germ cell tumours (TGCTs) are the most common malignancy of men in their 20’s and 30’s. TGCTs are clinically unique amongst metastatic malignancies in their exquisite sensitivity to platinum-based therapy such that advanced disease still carries a cure rate of approximately 80%^1^. Furthermore, TGCTs are also notable for the frequency of mixed histologic types on presentation, with seminoma as one major histology, versus the non-seminomatous histologies of embryonal carcinoma, yolk sac tumour, choriocarcinoma, and teratoma. While these various histologies are thought to derive from a common malignant stem cell, the ultimate lineage relationship amongst the components is still a matter of debate. Germ cell tumours by definition must derive from a stem cell since the healthy precursor tissue is the primordial germ cell (PGC), but whether the malignant components emerge in a linear fashion before or after the devolution to malignant disease is as yet unclear. In an era of genomically directed research and therapy, the possibility of diverse genetic signatures between different histologic components of disease carries significant clinical implications.

Post-pubertal TGCTs are known to carry pathognomonic gains of chromosome arm 12p along with near universal aneuploidy^2–6^. It is hypothesized that early germ cells undergo abnormal division that results in polyploidization followed by the formation of isochromosome 12p and invasion. *TP53* is nearly always wild type, which is a striking observation given that damaging mutations of *TP53* are the most common across all adult malignancies^7^. Amplification of *KIT* at 4q12 leading to overexpression as well as activating *KIT* mutations are well described, with these being most prevalent in seminomas^6,8,9^. The overall mutational burden in TGCTs is known to be relatively low at approximately 0.5 mutations per MB^10^ compared to the pan cancer rate of approximately 4.0 mutations per MB^11^. In addition, loss of heterozygosity (LOH) has been demonstrated^4,12^ with seminoma components of mixed tumours observed to have higher frequencies of LOH than pure seminomas^13^.

Data on the genomic characterization of germ cell neoplasia *in situ* (GCNIS) and of TGCTs, are accumulating, but most studies have characterized newly diagnosed TGCTs as a whole entity without parallel sequencing of the separate histologic components, or even specificity regarding which component was selected for analysis^4,14–17^. Recently, genomics, epigenomics, transcriptomics and proteomics have been utilized to assess different TGCT histological types present as the pure or major histological type in different patient tumours^6^. Our group previously leveraged Mate pair sequencing (MPseq) to identify genomic structural variants and to define lineage relationships in multifocal lung adenocarcinoma^18–21^. Here, we conduct parallel genomic characterization of distinct histological types within the same TGCT and between TGCTs from different patients for the purpose of defining the lineage relationships between different histologic populations.

## Results

Patient demographics and tumour samples are presented in Table 1. Different histological types were separated from each patient’s tumour with germ cell neoplasia *in situ* (GCNIS) representing the most commonly collected component (50% of the patients’ tumours), and with yolk sac tumour the least common (20% of the patients’ tumours) (Table 1). Details of the components collected from each patient can be found in Supplementary Table S1. Three tumours contained only one histological type (Patients 3, 6, and 10) while the others were mixed, containing 2–3 histologic types within the same tumour.

**Table 1:** Patient demographics.

Age range (median)	16–53 (28.5)
**Tumour types (n = 10)**	
Embryonal Carcinoma	2 (20%)
Seminoma	2 (20%)
Yolk Sac Tumour, Teratoma	2 (20%)
Mixed Embryonal Carcinoma, Seminoma	1 (10%)
Mixed Embryonal Carcinoma, Teratoma	1 (10%)
Mixed Embryonal Carcinoma, Yolk Sac Tumour	1 (10%)
Teratoma	1 (10%)
**Tumour components collected (from n = 10 tumours)**	
Germ Cell Neoplasia *in situ*	5 (50%)
Teratoma	4 (40%)
Embryonal Carcinoma	4 (40%)
Seminoma	3 (30%)
Normal Testicular Tissue	3 (30%)
Yolk Sac Tumour	2 (20%)

An average of 90 million fragments were obtained for each sample with an average of 91% concordant fragments (Supplementary Table S2). The average fragment size was 3700 base pairs. After filtering of junctions, which removed those with less than 7 supporting fragments, tumour samples were found to contain an average of 21 junctions per sample and normal samples (n = 3) an average of 5 junctions per sample. These normal tissue junctions were considered germline events and removed for lineage analysis, which tracks relatedness of samples using somatic junctions. After this germline junction filtering, germ cell neoplasia *in situ* possessed the fewest junctions per genome (median 1, range 0–4) and embryonal carcinoma the most (median 8.5, range 6–12) while yolk sac and teratoma each had a median of 6 junctions with a range of 2–10 and 3–13 respectively.

**Figure 1:**
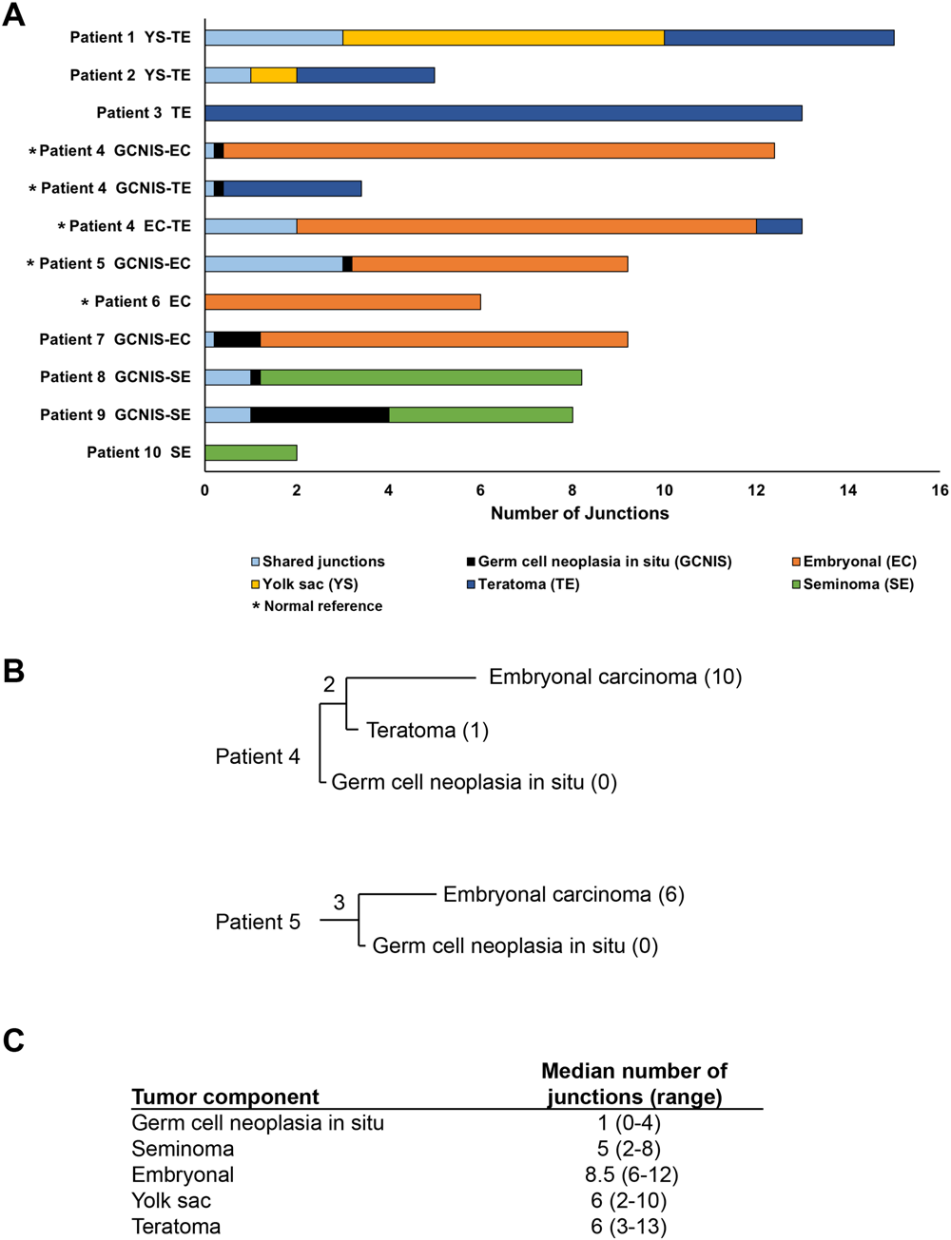
Shared and unique junctions present in different histologic types. (**A)** Number of shared and unique junctions found in different histologic types present in each patient’s tumour. Light blue boxes with a value <1 indicate no junctions shared by compared components and black boxes with value <1 indicate no junctions were unique to GCNIS. Patient 4 presented with three different tumour components, thus comparison between these components has been divided to accurately reflect shared and unique elements. **(B)** Dendrogram indicating lineage between components for patients 4 and 5. Numbers in parentheses indicate unique junctions, while numbers without parentheses indicate shared junctions. **(C)** Number of junctions observed in each tumour component.

The junctions reported in the samples showed that some tumours shared common junctions between the different histologic types, while others shared none (Fig. 1A). Three tumours contained a single histology (Fig. 1, Patients 3, 6 and 10) while seven tumours had mixed histologic types. Of the patients with normal tissue available for sequencing (patients 4, 5 and 6), only patients 4 and 5 had more than one histologic type collected in addition to normal tissue. Germline junctions allowed the assessment of background junctions that are not unique to the tumour or its histologic types. Furthermore, the presence of germline data can confirm the samples came from the same patient tissue, thus serving as an internal control in the absence of other lineage markers. While germline junctions were observed in each of the normal patient samples, none of these germline junctions were shared between the normal patient samples of patients 4, 5, and 6.

The relationships of junctions between components from the same patient are seen in Fig. 1B with details of the observed junctions in Supplementary Table S3. There were 144 interchromosomal and 117 intrachromosomal junctions detected across all samples. The embryonal cell populations had the largest shift towards interchromosomal with 67 interchromosomal junctions detected vs. 26 intrachromosomal junctions detected. The other histologies were either split evenly between intra- and interchromosomal or were mostly intrachromosomal. A slight majority of the intrachromosomal junctions were <300 kb (50.4%) and likely germline with an overall median size of 270Kb. Interestingly, the GCNIS in patient 5 shared a common lineage with the associated embryonal carcinoma, but in contrast, the GCNIS from patient 4 shared no common lineage with the associated embryonal carcinoma and teratoma, although the latter two shared a common lineage. Similar to patient 4, the GCNIS component of patient 7 shared no common lineage with its embryonal carcinoma component. In contrast, the GCNIS components of patients 8 and 9 both shared a common lineage with their seminoma components. Of note, the only component lacking detectable junctions in a subset of samples was the GCNIS.

**Figure 2:**
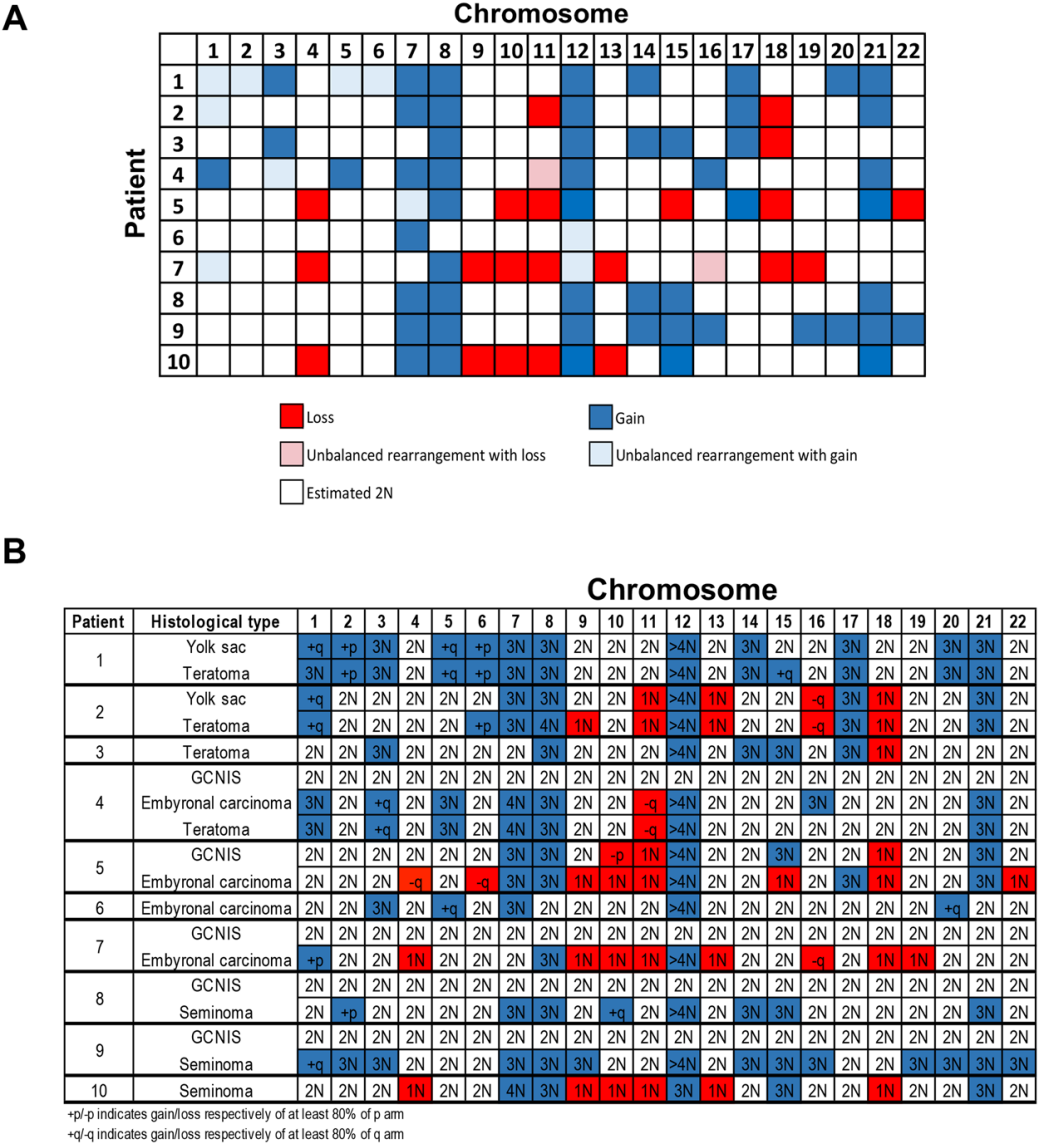
Copy number variations. (**A)** Global aneuploidy and chromosomal instability across chromosomes and patient samples. **(B)** Aneuploidy of specific histological types across chromosomes and patient samples.

**Figure 3:**
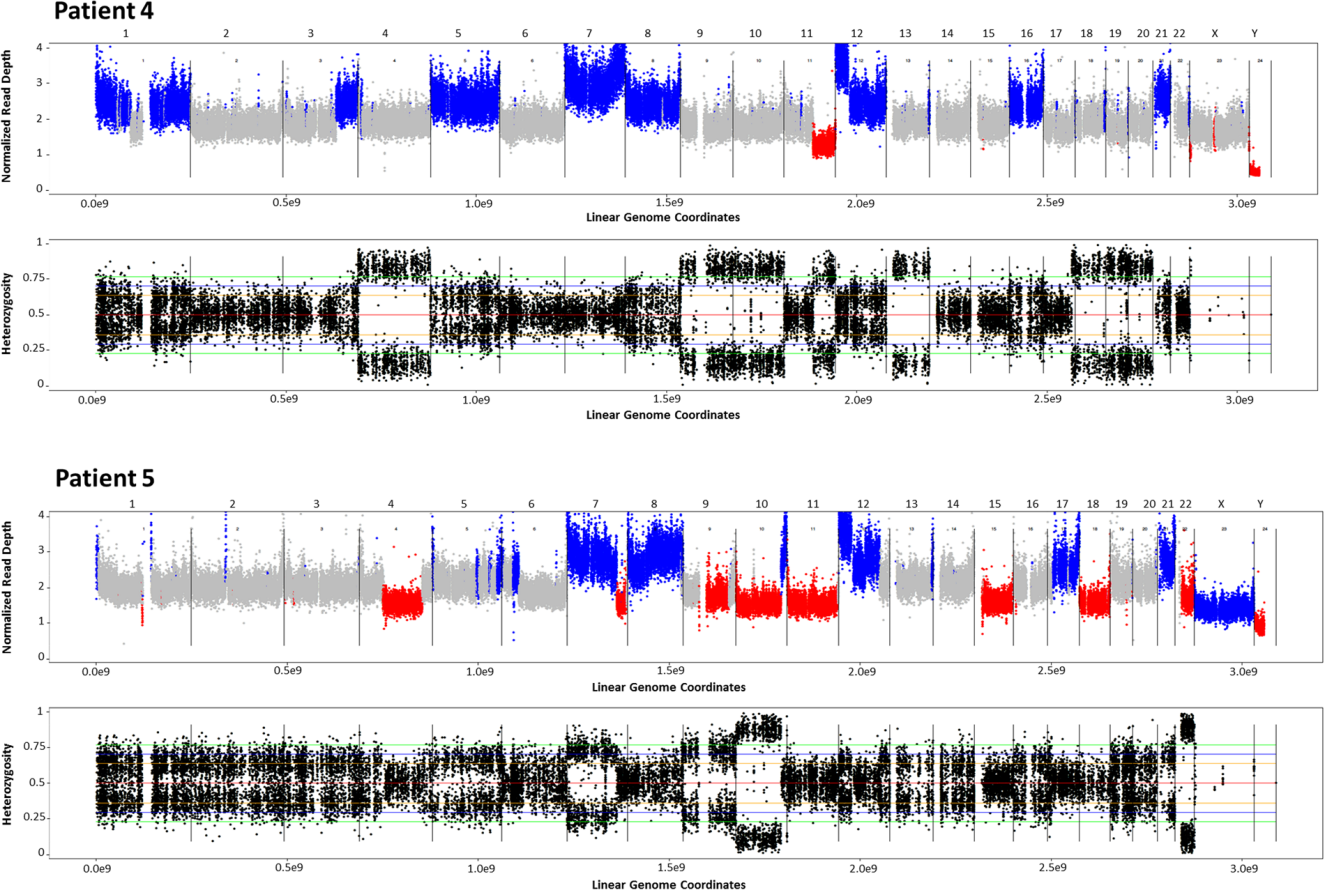
Copy neutral loss of heterozygosity in patients 4 and 5. Top image presents the normalized read depth. Blue indicates copy number gain, red copy number loss, and grey indicates the region is at the expected 2N level. Bottom image shows allelic percentage for positions identified as SNPs in the normal sample. Areas of the genome with LOH or structural loss or gain will show deviation from the expected allelic percentage (=0.5) for the reference nucleotide.

Analysis of the samples for common copy number variant (CNV) features revealed a considerable aneuploidy with whole-arm or whole-chromosome gains or losses, demonstrating chromosomal instability across the samples (Figs 2 and S1). With the exception of GCNIS, which show little variation in general, this aneuploidy was observed in all histologies and most often included chromosomal gains. The only CNV observed in all histological types and across patient tumours was a multiple gain (3N–5N) in chromosome arm 12p, which is a known pathognomonic feature of post-pubertal TGCTs that we detected in all 10 cases (Fig. 2). Of note, the 12p gain was not observed in four of the five GCNIS samples, but was present in adjacent embryonal carcinoma, seminoma or teratoma present in the same tumour (Figs 3B and S2). Additionally, gains in chromosomes 7, 8, and 21 were observed in 7/10 cases. We did not observe a single case with an amplification of *KIT* at 4q12, which has been described in 17–21% of seminomas^9,22^ and 9% of nonseminomas^9^. In addition to the shared 12p gain observed globally, when CNV were compared between particular histological types additional shared CNV were observed. No shared CNV were observed between the GCNIS and seminoma histologic types. While patient 5 had shared copy number gains in chromosomes 7, 8 and 21 as well as shared copy number loss in chromosomes 10, 11 and 18 between GCNIS and embryonal carcinoma, patients 4 and 7 did not share CNV between the GCNIS and embryonal carcinoma. Yolk sac and teratoma shared gains in chromosomes 1, 7, 8, 17, and 21 in patients 1 and 2 as well as copy number loss for patient 2 in chromosomes 11, 13, 16 and 18. Patient 4 whose tumour possessed embryonal carcinoma as well as teratoma shared copy number gains in chromosomes 1, 3, 5, 7, 8, and 21 as well as loss of 11q in these components.

For further expansion of the genomic relationships between histologically distinct tumour components described through the MPseq analysis, Exome sequencing (ExomeSeq) was performed in the GCNIS and embryonal carcinoma from two of the cases (Patients 4 and 5). As observed in MPseq, the GCNIS component for Patient 5 shared 7 somatic mutations with the embryonal carcinoma (Supplementary Table S4). This demonstrated a close relationship between the two histologically distinct tumour components. In contrast for patient 4, there were no shared junctions between the embryonal carcinoma and the GCNIS and only one shared missense mutation.

In addition to the mutational analysis, the ExomeSeq data enabled an analysis of copy-neutral loss of heterozygosity (cnLOH) events where the physical copy number of a chromosome remains at 2 N, but both copies contain the same allele. For Patient 5 there were two whole-chromosome cnLOH events on chromosomes 10 and 22 (Fig. 3). For Patient 4 there were six whole-chromosome cnLOH events on chromosomes 4, 9, 10, 18, 19, and 20. The high level of cnLOH in these two patients is consistent with recent reports in the literature.

## Discussion

**Figure 4:**
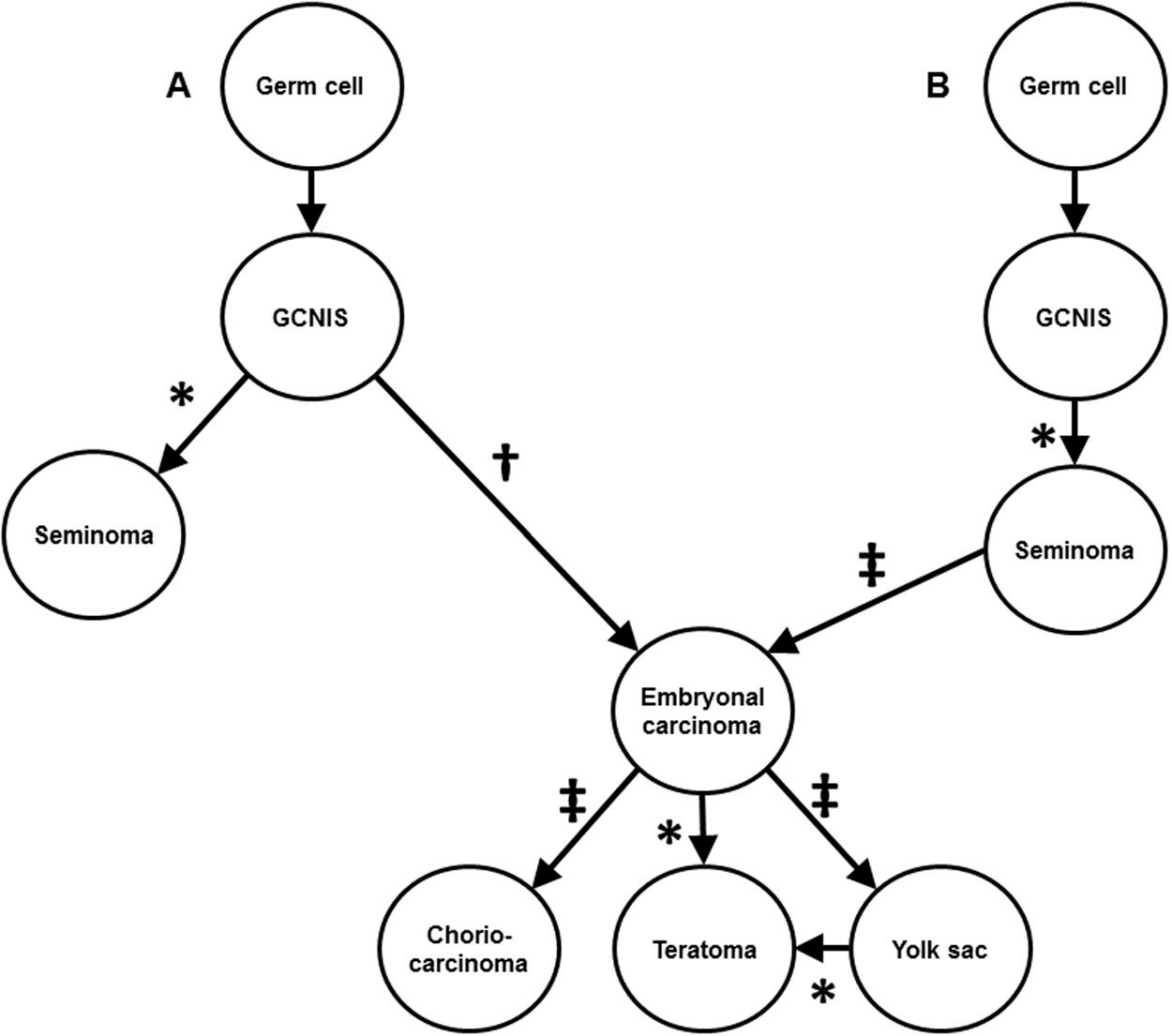
Models of germ cell tumour evolution with evidence from this study. Germ cell neoplasia *in situ* (GCNIS) develops from a normal germ cell. However, the exact genomic events that lead to GCNIS and subsequent invasive TGCT are not well understood. It is believed that seminoma and embryonal carcinoma may derive directly from GCNIS **(A)**, or embryonal carcinoma may derive in a linear fashion from seminoma **(B)**^1,25,27,28,40,41^. Teratoma, yolk sac and choriocarcinoma histological types have been demonstrated to arise from embryonal carcinoma^1,25,27–30^. Symbols: *study evidence supports this relationship, ^†^study evidence inconclusive, ^‡^study unable to assess relationship.

In some ways, TGCTs with mixed histology represent a visible manifestation of the universal issue of tumour clonality. TGCTs have the advantage that the phenomenon of clonality is already integrated into the treatment paradigms, with risk classification and the use of radiation therapy being dependent on histology^23^. Furthermore, the World Health Organization (WHO) recommends the use of the term germ cell neoplasia *in situ* (GCNIS) for precursor lesions of invasive germ cell tumours and established a classification of testicular tumours based on whether a tumour originated from GCNIS or not because dividing tumours based solely on morphological characteristics could place tumours with very different pathogeneses in the same class^24^. GCNIS-derived tumours share similar morphologic characteristics and amplification of chromosome arm 12p^24^. Previous data suggests that while seminoma can arise directly from GCNIS, the non-seminoma histologic types, teratoma, yolk sac tumour, and choriocarcinomas arise from embryonal carcinoma^25^. We contend that parallel analysis of the different components of TGCTs represents a logical extension of characterizing TGCTs by their various histologic components for at least two reasons. First, lineage analyses can help validate the competing models of development of TGCTs (Fig. 4). Second, molecular classifiers of cancer are now being incorporated into clinical guidelines for other cancers, for example RAS/RAF and microsatellite instability (MSI) testing in colon cancer^26^. We hypothesize that molecular classification of TGCTs holds the potential to better identify treatment resistant (and thus potentially life threatening) TGCTs. If the hypothesis is correct, then parallel analysis of different histologic components is likely to be undeniably important since a single treatment resistant clone will necessarily drive clinical outcomes over any mass of treatment sensitive clones.

Our data supports the early divergence of the different histological components in testicular germ cell tumours with the accumulation of structural variants, including the 12p isoform, and increasing mutation burden in the invasive component. Our junction data supports the common lineage of GCNIS and seminoma histologic types (patients 8 and 9) (Fig. 4). While embryonal carcinoma derivation from GCNIS has been documented^1,25,27–29^, whether it can derive directly from GCNIS or via seminoma remains to be determined^25^. Tumours from patients 4, 5 and 7 all possessed GCNIS and embryonal carcinoma, but only patient 5’s tumour had shared junctions between these components, as well as copy number loss while tumours from patients 4 and 7 do not possess any shared junctions or CNVs between GCNIS and embryonal carcinoma. However, the ExomeSeq data from patient 4 identified a single, shared missense mutation in the RELN gene between GCNIS and embryonal carcinoma. Thus, the evidence supporting the lineage between GCNIS and embryonal carcinoma is inconclusive in this cohort (Fig. 4). Tumours with seminomatous components (patients 8 and 9) had no embryonal carcinoma present, thus potential lineage of embryonal carcinoma from seminoma could not be assessed (Fig. 4). In contrast, the lineage of embryonal carcinoma and teratoma is well established^1,25,27,28,30^, and this shared lineage was observed in the only tumour (patient 4) in this cohort with embryonal carcinoma and teratoma components (Fig. 4). A possible shared lineage between yolk sac and teratoma has been suggested^1^, and is supported by this data as tumours from patients 1 and 2 contained yolk sac and teratoma histologies with shared junctions and CNV.

Despite the model predicting linear evolution from GCNIS to embryonal carcinoma and more aggressive components, it is common that precursor components can be missing from mixed nonseminomatous TGCT. Tumours from patients 8 and 9 had GCNIS and seminoma components while patient 10 had only seminoma without evidence of GCNIS. One tumour with only teratoma (patient 3) and two tumours containing yolk sac and teratoma (patients 1 and 2) did not contain an embryonal carcinoma in the tumour. It is not clear from this data whether these intermediate steps can be bypassed, if they are present in undetected low volumes, or have previously regressed.

Junctions with one or both breakpoints hitting gene locations were compared against the Catalog of Somatic Mutations in Cancer (COSMIC) database^31^ to identify potentially deleterious events. Only four junctions fell within genes in the COSMIC gene list. Each junction had <7 supporting reads which fell below the cut-off of 7 supporting fragments used for determining high quality junctions in lineage analysis. Thus, analysis of these junctions was not conducted. Close breakpoints such as deletions and tandem duplications, occur commonly within human germline genomes and are generally considered innocuous, thus these were excluded from analysis in the absence of a normal control. No known tumour suppressors were determined to have been impacted by the presence of junctions or copy number changes in this dataset which raises the question of whether this avoidance of alterations in tumour suppressors could contribute to the less aggressive nature of TGCTs.

The interpretation of the data is limited by the heterogeneity of the histological types present in the tumours, limited availability of normal samples, and the small sample size. In addition, ExomeSeq could only be conducted on samples from two patients, thus the data was unable to capture copy number trends in LOH between the histologic types studied in this work. A larger sampling of tumours sharing the same histological components and with accompanying normal samples for comparison may have rendered more definitive lineage associations to support or refute the currently accepted lineage model.

Understanding the relationship between primary tumours and chemorefractory recurrent disease can help develop rationale hypotheses for clinically impactful investigation. Identification of prognostic and predictive markers remains a major clinical goal of genomic analyses. The current approach to the management of recurrent TGCTs is to continue to rely on platinum based chemotherapy combinations, with later escalation to more aggressive regimens for relapsed disease, including the use of high dose chemotherapy and autologous stem cell transplantation in the second or in the third line^23^. Consequently, identification of markers of resistance to first line therapy may provide a rational basis for early intensification of therapy, whether with high dose chemotherapy or other regimens, if a biomarker could be tested for in the primary tumour. The variability observed between histological types within the same tumour in this dataset supports the idea that genomic characterization of only one component of mixed TGCTs will necessarily miss additional abnormalities present elsewhere in the tumour. The lack of definitive linearity in evolution also precludes a strategy of simply characterizing any single histology with the idea that it represents the latest step in a linear evolution which would thus be inclusive of all accumulated genetic aberrations. Since prediction of chemoresistance is dependent on the detection of the chemoresistant cell population, even a valid predictive biomarker could fail if a patient has chemoresistant disease in an unanalysed portion of his tumour.

In conclusion, this work demonstrates that many unique genomic structural variants are present in different histological tumour components and these structural variants differ between patients. Further study of testicular germ cell lineage is needed to better define the disease evolution and how that may impact treatment decisions.

## Methods

### Patient selection and sample collection

Informed consent was obtained for tissue collection under the Biomarker Discovery Program Frozen Tissue Collection protocol (IRB# 17-001218) and approved for use by the Mayo Clinic Institutional Review Board as part of the Genitourinary Clinical Genomics Project (IRB# 15-001429). All available samples of testicular GCTs in the Frozen Tissue Collection were utilized for this protocol. Adjacent normal tissue was available and collected in Patients 4, 5 and 6. Methods were carried out in accordance with the relevant institutional guidelines.

### Isolation of DNA and Mate-Pair Sequencing

**Figure 5:**
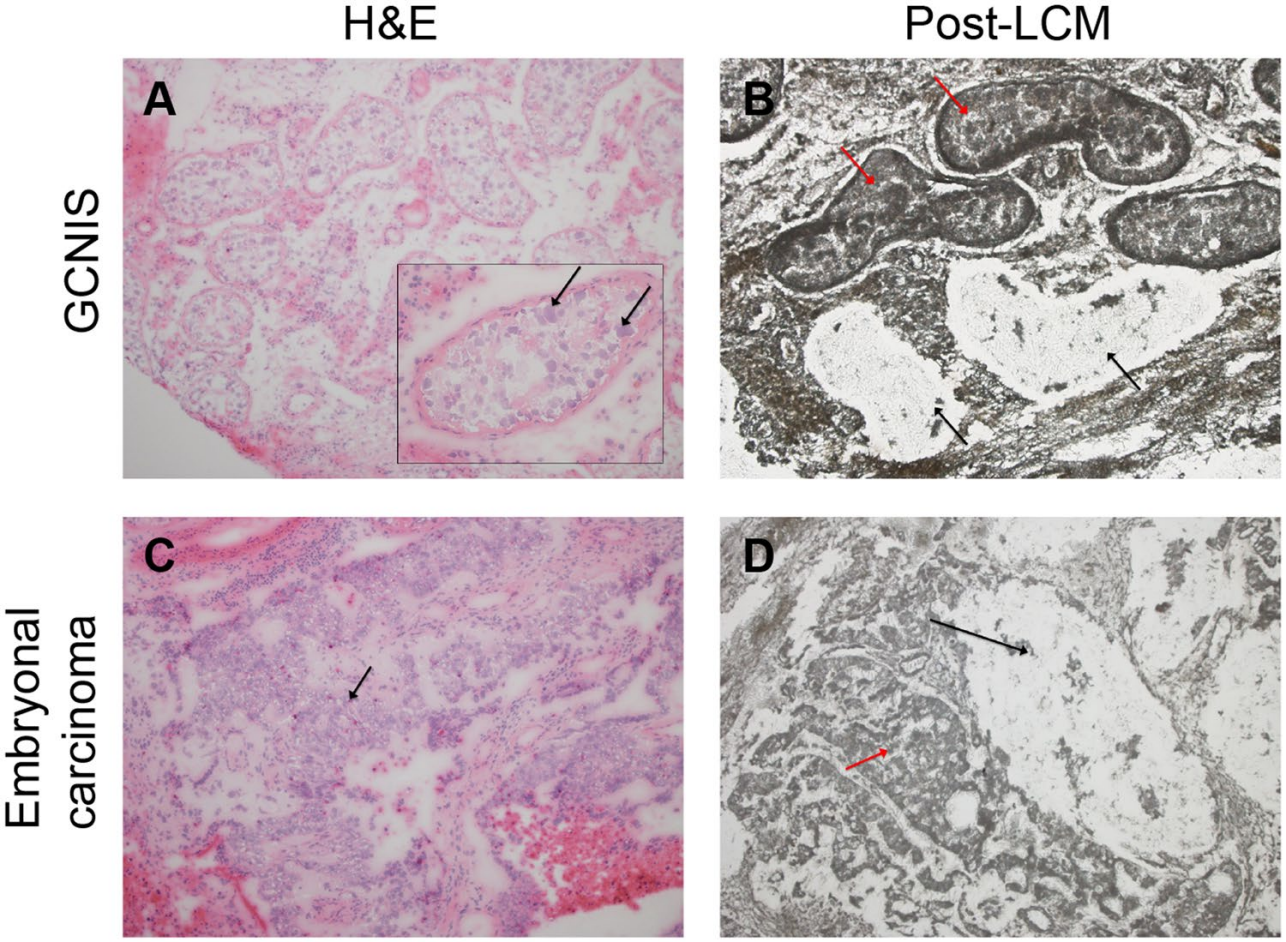
Representative images of tissues and their associated pathology. (**A)** Haematoxylin and Eosin (H&E) stain of frozen GCNIS tissue. Arrows indicate GCNIS with large neoplastic cells within the tubules. **(B)** Unstained GCNIS tissue section following laser capture microdissection (LCM). Black arrows indicate tubules with GCNIS captured by LCM. Red arrows indicate intact tubules. **(C)** H&E stain of frozen embryonal carcinoma tissue indicated by arrow. **(D)** Unstained embryonal carcinoma tissue post-LCM. Black arrow indicates captured region while red arrow indicates intact tumour.

The tissue was first reviewed by a urologic pathologist (JC) for histology and tumour content and the histologic components were isolated using laser capture microdissection (LCM) (Arcturus instrument) from unstained fresh-frozen tissues sections (10 μm). Representative images of tissues and their associated pathology are presented in Fig. 5. DNA was amplified directly from captured cells using a previously described modified Repli-g protocol^18–21^. Indexed libraries were prepared from the whole genome amplified DNA using the Illumina Mate-Pair (MP) Kit following the manufacturer’s instructions and sequenced as two libraries per lane on the Illumina HiSeq2000 platform (2 × 101bp)^18–21^.

### Structural Variant Analysis

The BMD Structural Variant Pipeline (BMD SV Pipeline) analyses Mate-Pair sequencing (MPseq) data to identify and report structural variants. The structural variants reported include: (1) junctions of chromosomal rearrangements greater than 30 kb, with the junctions identifying the reunion of the breaks occurring in chromosomal rearrangements such as deletions, inversions, or translocations and (2) copy number variants (CNVs). Analysis is a two-step process: mapping MPseq fragments and structural variant calling. Sequencing data was mapped to the reference genome GRCh38 using the binary indexing mapping algorithm, BIMA^32^, and passed to structural variant analysis with SVAtools. SVAtools consists of a junction detection step^33^, a CNV detection step^34^, followed by integration of the results from both steps to improve detection sensitivity and breakpoint resolution. The junction detection step returns the two breakpoints of a junction and the number of MPseq fragments supporting each junction. For lineage assessment, a quality threshold of ≥7 supporting MPseq fragments was applied for filtering reported junctions. If a junction was identified with ≥7 supporting MP fragments, then the other samples were manually checked for the presence of this junction with at least 3 supporting fragments. Additionally, in the absence of a normal control, intrachromosomal junctions were required to be larger than 50Kb to avoid common polymorphisms such as insertion-deletions, and duplications. If the intrachromosomal junctions were between 50Kb and 100Kb, then they were manually investigated to determine if they fell in known genomic regions or in non-genic regions. Those falling in non-genic regions were removed. Junctions from different samples were considered the same if the distances between both breakpoints of the junction were within 10Kb. After filtering, lineage was assessed by comparing the shared and unique junctions of each histological type within a single tumour, excluding junctions present in the normal tissue. The CNV detection step segments the genome and determines whether there is a loss or gain of genetic material in each segmented region. These losses and gains are either a result of a chromosomal rearrangement and have a corresponding supporting junction or result from a whole-arm or whole-chromosome gain or loss and no junction is present. The whole genome data was visualized using the Genome U-Plot^35^.

### Exome Sequencing

Exome sequencing (ExomeSeq) was performed on the same whole genome amplified DNA as utilized for MPseq from each histological component isolated from two cases. ExomeSeq libraries were sequenced as four libraries per lane on the Illumina HiSeq2000 platform (2 × 101bp). In this study, the Mayo Clinic GenomeGPS 4.0 pipeline was utilized for exome data analysis. It includes three steps: alignment, single nucleotide variant (SNV) and small insertion/deletion (Indel) variant calling, and structural variation annotation. All tools were run under default configuration unless otherwise specified. FASTQ files were aligned to the GRCh38 reference genome using BWA 0.7.10 with the BWA-MEM algorithm for short read alignment^36,37^. Realignment was performed using GATK Version 3.4^38^ and all variants reported. Variant coding regions were functionally annotated using the BioR Version 2.5 annotation engine developed at Mayo Clinic^39^. Coverage limits of ≥20 supporting reads in both normal and tumour samples were used to remove mutations that were not sufficiently covered in the sequencing as well as the removal of mutations that did not impact the final protein product (intronic, untranslated, and synonymous mutations), as these are more likely sources of false positives than mutations that do impact the final protein product.

## Supplementary information

Supplementary data

## Data Availability

The data generated and analysed during the current study are available in the Sequence Read Archive (SRA) repository, https://www.ncbi.nlm.nih.gov/bioproject/PRJNA523142 .
