# Supplementary material for: Shared and unique genomic structural variants of different histological components within testicular germ cell tumours identified with mate pair sequencing
Source: Sci Rep. 2019 Mar 5;9:3586. doi: 10.1038/s41598-019-39956-y (PMC6400951; doi:10.1038/s41598-019-39956-y)
Supplement: Supplementary file 1 — Supplementary data [file 41598_2019_39956_MOESM1_ESM.pdf]

# Shared and unique genomic structural variants of different histological components within testicular germ cell tumors identified with mate pair sequencing


Alan H. Bryce[1,2,3*], Jan B. Egan[3], James B. Smadbeck[3], Sarah H. Johnson[3], Stephen J. Murphy[3], Faye R. Harris[3], Geoffrey C. Halling[3], Simone B. S. P. Terra[4], John Cheville[4], Lance Pagliaro[5], Brad Leibovich[6], Brian A. Costello[5], George Vasmatzis[3,7]

**Affiliations:**

[1] Division of Hematology/Oncology Mayo Clinic, Phoenix, Arizona, USA

[2] Mayo Clinic Cancer Center, Phoenix, Arizona, USA

[3] Center for Individualized Medicine, Mayo Clinic, Rochester, Minnesota, USA

[4] Department of Laboratory Medicine and Pathology, Mayo Clinic, Rochester, Minnesota, USA

[5] Department of Medical Oncology, Mayo Clinic, Rochester, Minnesota, USA

[6] Department of Urology, Mayo Clinic, Rochester, Minnesota, USA

[7] Department of Molecular Medicine, Mayo Clinic, Rochester, Minnesota, USA

*Corresponding author:

Alan H. Bryce, M.D.

Email: bryce.alan@mayo.edu

**Supplementary Figure S1.** Genome Plot of abnormal junctions color coded by Patient Id. The chromosomes are arranged in a u-shape configuration. The endpoints of each line indicate the breakpoints of the junction. The breakpoints hit randomly throughout the genome and vary by patient. Junctions are similar only within the patient histological sub-types. See Supplementary Table 3 for a detailed list of these junctions.

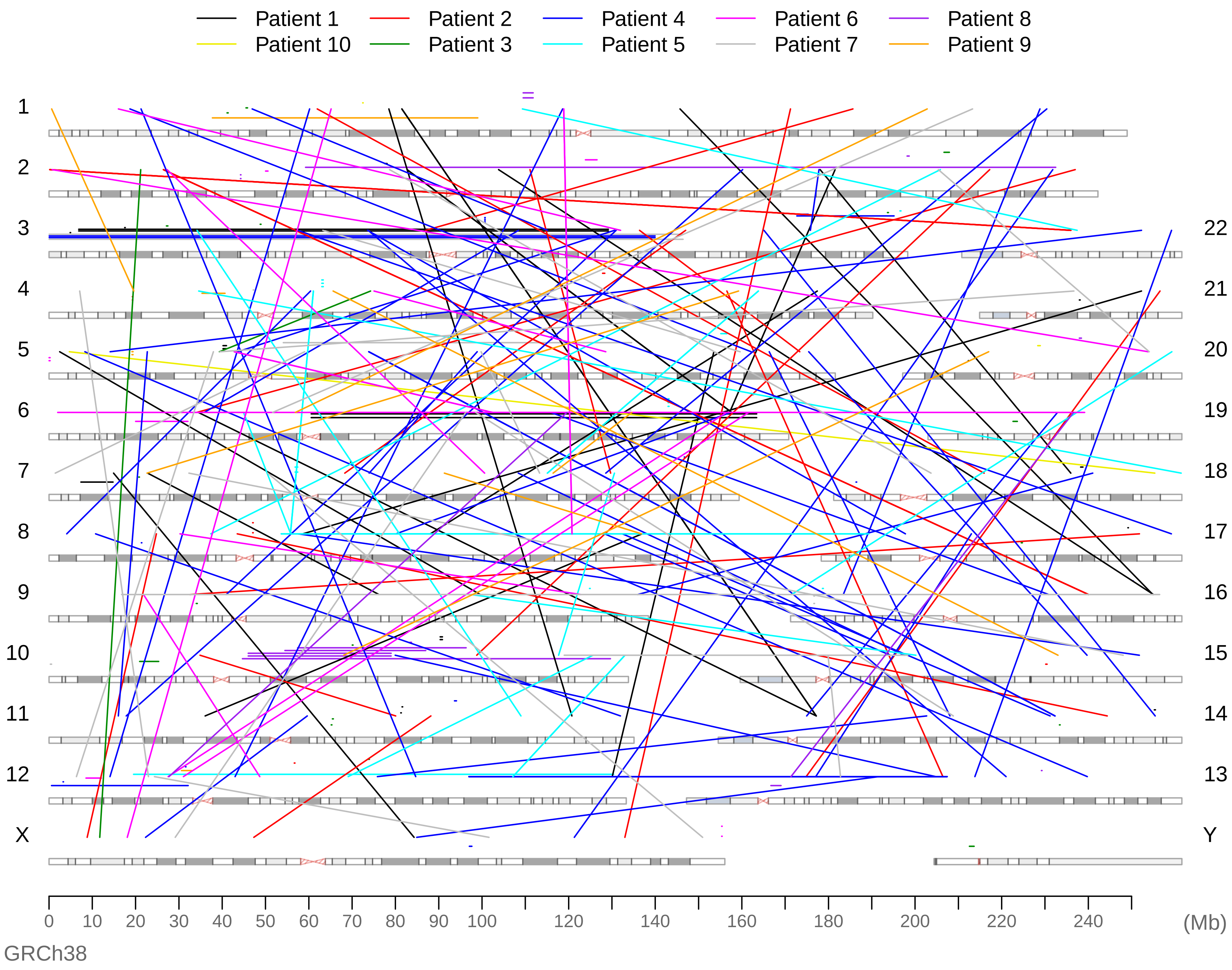
Patient 1
Patient 2
Patient 4
Patient 6
Patient 8
Patient 10
Patient 3
Patient 5
Patient 7
Patient 9
1
2
3
4
5
6
7
8
9
10
11
12
X
22
21
20
19
18
17
16
15
14
13
Y
0
10
20
30
40
50
60
70
80
90
100
120
140
160
180
200
220
240
(Mb)
GRCh38

**Supplementary Figure S2.** Copy number variants observed in each histological type by patient. The graphs are normalized read depth. Blue indicates copy number gain, red copy number loss, and gray indicates the region is at the expected 2N level.

# Patient 1

## Yolk sac

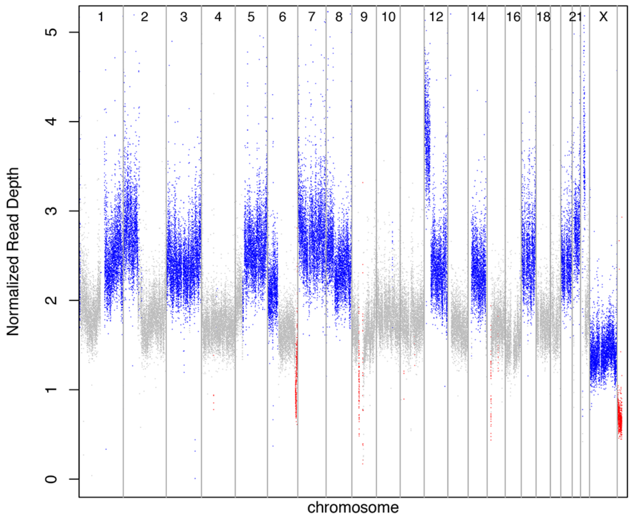


## Teratoma

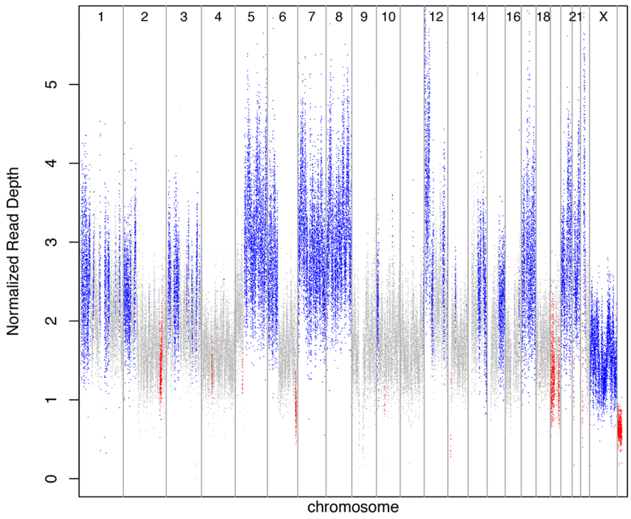

# Patient 2

## Yolk sac

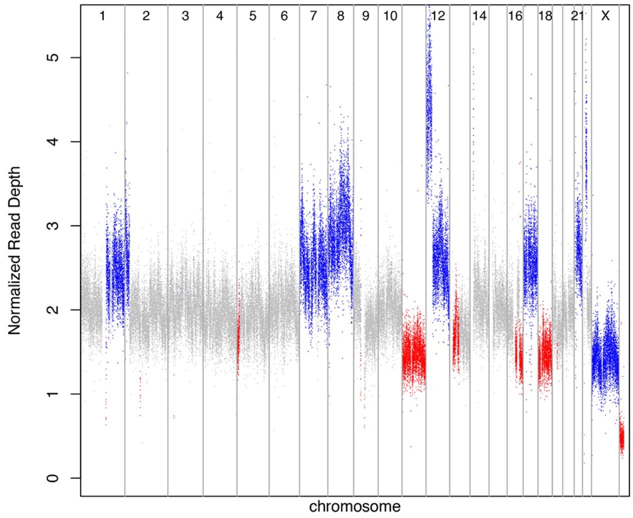


## Teratoma

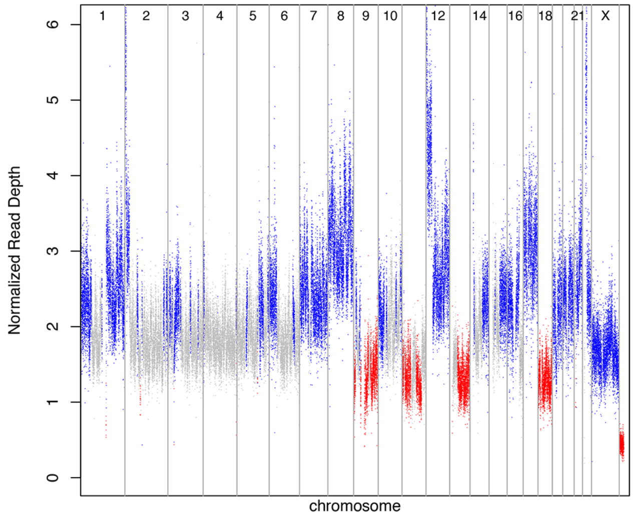

# Patient 3

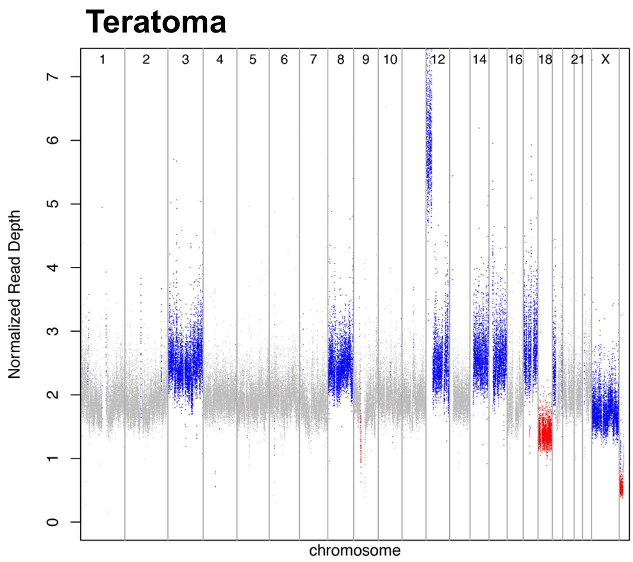

Teratoma
1
2
3
4
5
6
7
8
9
10
12
14
16
18
21
X
Normalized Read Depth
0
1
2
3
4
5
6
7
chromosome

# Patient 4

## Germ cell neoplasia in situ

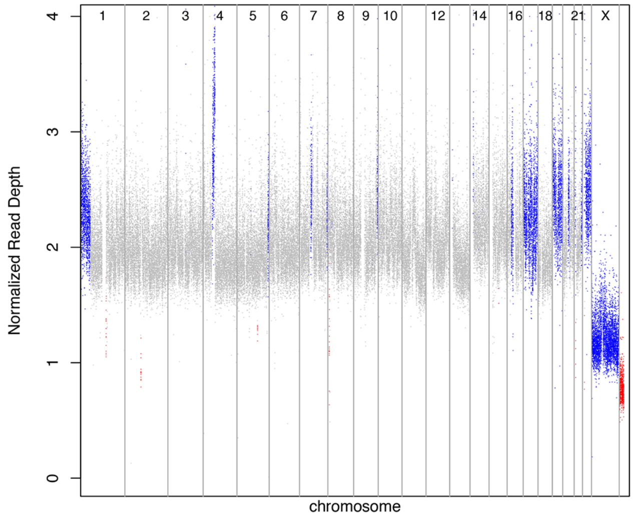


## Embryonal carcinoma

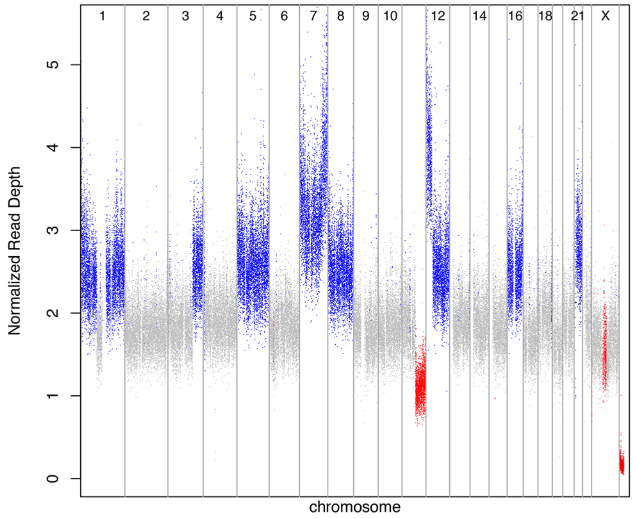


## Teratoma

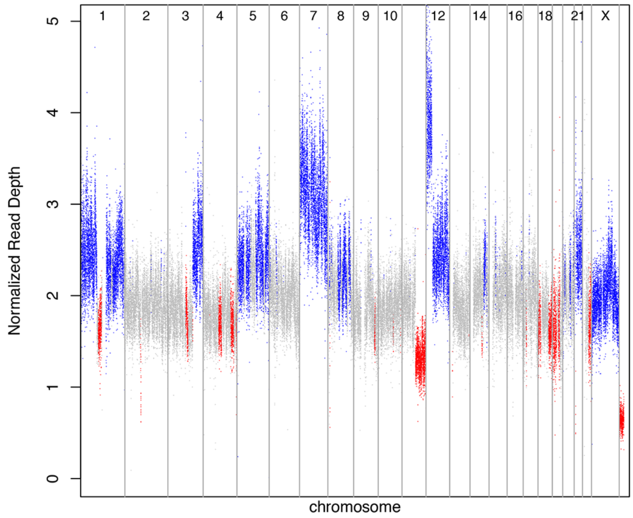

# Patient 5

## Germ cell neoplasia in situ

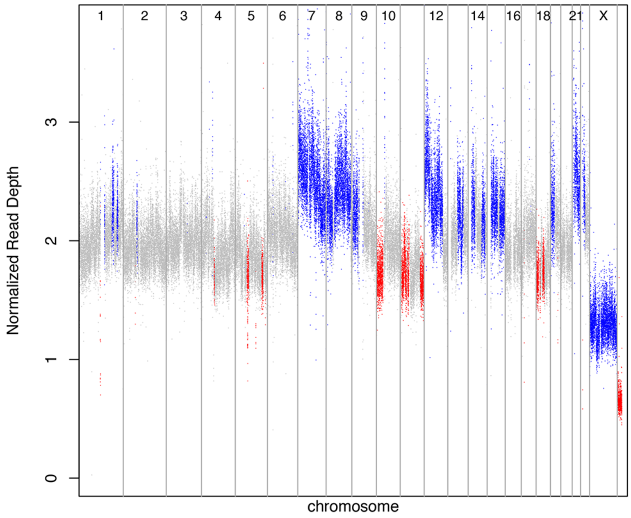


## Embryonal carcinoma

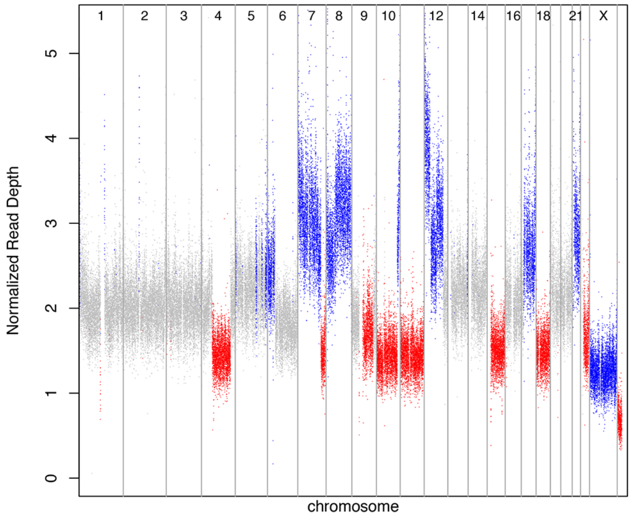

# Patient 6

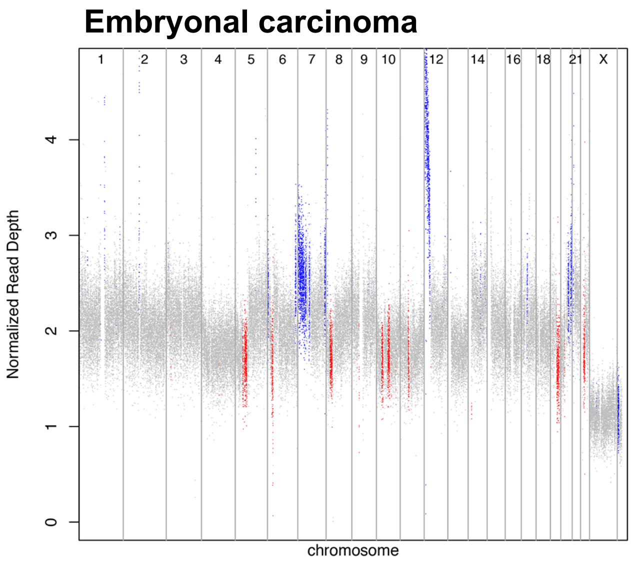
Embryonal carcinoma
Normalized Read Depth
0
1
2
3
4
1
2
3
4
5
6
7
8
9
10
12
14
16
18
21
X
chromosome

# Patient 7

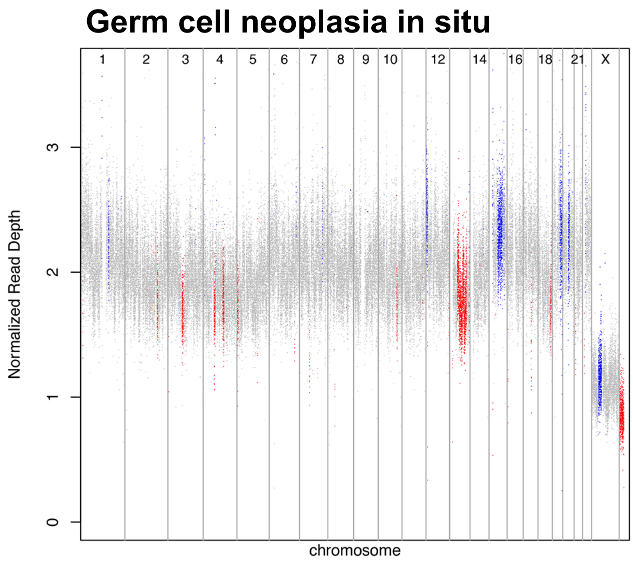
Germ cell neoplasia in situ
1
2
3
4
5
6
7
8
9
10
12
14
16
18
21
X
0
1
2
3
Normalized Read Depth
chromosome

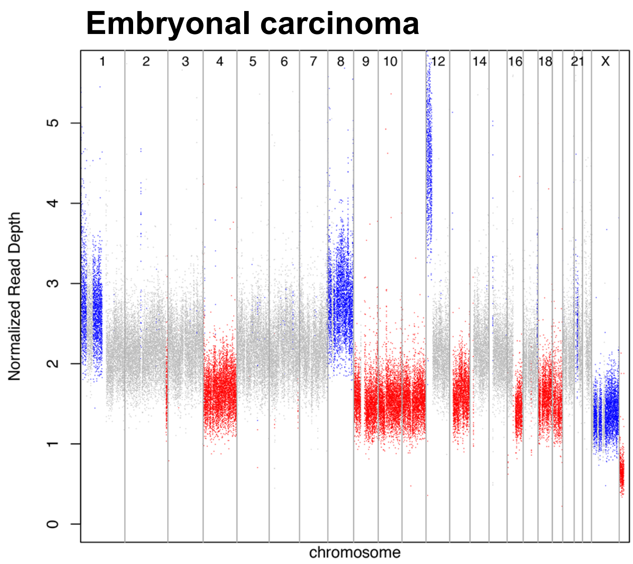
Embryonal carcinoma
1
2
3
4
5
6
7
8
9
10
12
14
16
18
21
X
0
1
2
3
4
5
Normalized Read Depth
chromosome

# Patient 8

## Germ cell neoplasia in situ

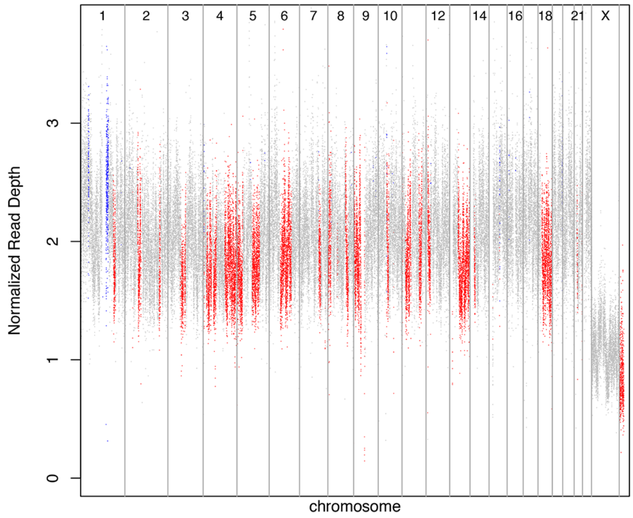


## Seminoma

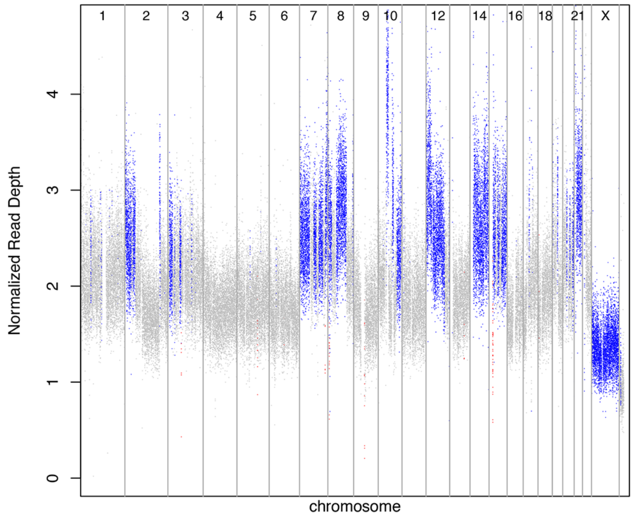

# Patient 9

## Germ cell neoplasia in situ

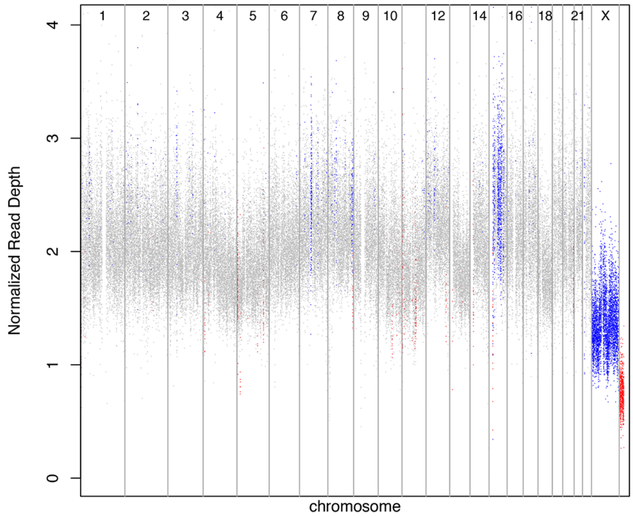


## Seminoma

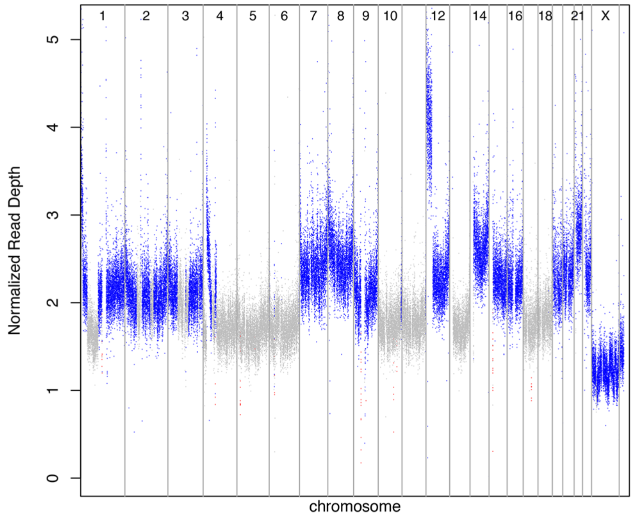

# Patient 10

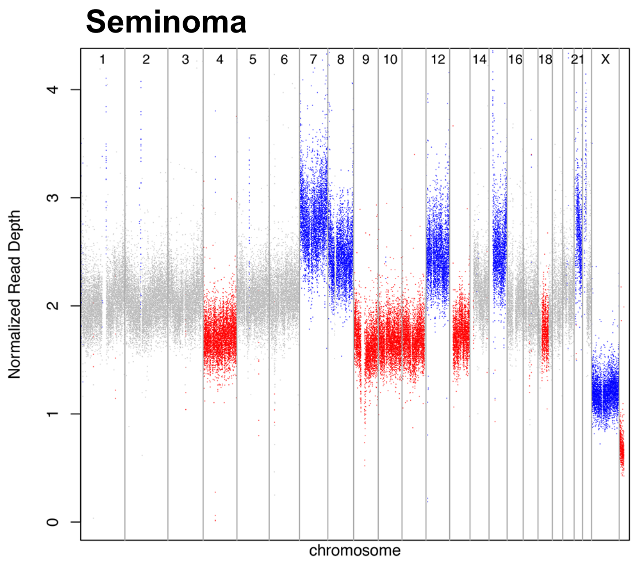
Seminoma
1
2
3
4
5
6
7
8
9
10
12
14
16
18
21
X
Normalized Read Depth
0
1
2
3
4
chromosome

**Supplementary Tables summary**

**Supplementary Table S1.** Summary of tumor components collected for each patient.

**Supplementary Table S2.** Sequencing metrics.

**Supplementary Table S3.** Summary of chromosomal rearrangements.

**Supplementary Table S4.** Summary of insertion/deletions and single nucleotide variants.

**Table S1. Summary of Tumor Component Collected for Each Patient**

| Patient | Normal | Germ cell neoplasia in situ | Seminoma | Embryonal | Teratoma | Yolk sac |
|---|---|---|---|---|---|---|
| 1 | | | | | | |
| 2 | | | | | | |
| 3 | | | | | | |
| 4 | | | | | | |
| 5 | | | | | | |
| 6 | | | | | | |
| 7 | | | | | | |
| 8 | | | | | | |
| 9 | | | | | | |
| 10 | | | | | | |

Dark green shading indicates tissue component collected

**Table S2. Sequencing metrics**

| Sample | Read Length | Total Fragments | Mapped Fragments | % Mapped Fragments | Replicate Fragments | % Replicate Fragments | % Concordant | % Discordant | Fragment Size (mode) | Fragment Size (mean) | Bridged Coverage (mode) | Bridged Coverage (mean) | Base Coverage (mode) | Base Coverage (mean) |
|---|---|---|---|---|---|---|---|---|---|---|---|---|---|---|
| Patient 1 teratoma | 101 | 99,188,305 | 99,053,899 | 99.86% | 3,039,007 | 3.06 | 90.54 | 4.16 | 2230 | 3698 | 59 | 90 | 1 | 4 |
| Patient 1 yolk sac | 101 | 85,731,646 | 85,627,451 | 99.88% | 2,633,762 | 3.07 | 90.77 | 3.89 | 2223 | 3682 | 62 | 78 | 1 | 3 |
| Patient 2 teratoma | 101 | 85,937,481 | 85,816,076 | 99.86% | 2,350,611 | 2.74 | 90.94 | 4.33 | 2078 | 3750 | 48 | 79 | 1 | 3 |
| Patient 2 yolk sac | 101 | 86,436,431 | 86,334,217 | 99.88% | 3,002,146 | 3.47 | 90.82 | 3.58 | 2192 | 3895 | 62 | 83 | 1 | 3 |
| Patient 3 teratoma | 101 | 88,983,358 | 88,876,420 | 99.88% | 2,440,207 | 2.74 | 90.51 | 4.56 | 2202 | 4092 | 70 | 82 | 2 | 3 |
| Patient 4 embryonal carcinoma | 101 | 88,163,663 | 88,073,558 | 99.90% | 2,695,617 | 3.06 | 90.29 | 4.50 | 2234 | 3716 | 57 | 79 | 1 | 3 |
| Patient 4 germ cell neoplasia in situ | 101 | 83,845,625 | 83,749,105 | 99.88% | 2,267,621 | 2.70 | 90.95 | 3.95 | 2359 | 3700 | 66 | 78 | 2 | 3 |
| Patient 4 teratoma | 101 | 104,571,029 | 104,458,001 | 99.89% | 3,134,768 | 3.00 | 90.62 | 4.12 | 2241 | 3654 | 67 | 93 | 2 | 4 |
| Patient 4 normal | 101 | 90,446,288 | 90,344,576 | 99.89% | 2,322,201 | 2.57 | 90.97 | 4.33 | 2286 | 3774 | 65 | 84 | 2 | 3 |
| Patient 5 embryonal carcinoma | 101 | 102,291,364 | 102,172,964 | 99.88% | 2,898,172 | 2.83 | 90.67 | 4.63 | 2003 | 3556 | 62 | 89 | 1 | 4 |
| Patient 5 germ cell neoplasia in situ | 101 | 105,773,175 | 105,638,154 | 99.87% | 3,279,944 | 3.10 | 90.38 | 4.22 | 2233 | 3860 | 78 | 98 | 2 | 4 |
| Patient 5 normal | 101 | 67,852,570 | 67,771,781 | 99.88% | 1,422,490 | 2.10 | 91.00 | 4.37 | 2144 | 3903 | 63 | 67 | 1 | 3 |
| Patient 6 embryonal carcinoma | 101 | 70,281,707 | 70,171,454 | 99.84% | 1,730,947 | 2.46 | 91.62 | 3.87 | 1811 | 3185 | 44 | 59 | 1 | 3 |
| Patient 6 normal | 101 | 81,637,812 | 81,508,764 | 99.84% | 2,130,014 | 2.61 | 91.27 | 3.91 | 1996 | 3485 | 56 | 71 | 1 | 3 |
| Patient 7 embryonal carcinoma | 101 | 95,565,590 | 95,393,032 | 99.82% | 5,474,277 | 5.73 | 88.19 | 4.07 | 2027 | 3208 | 49 | 73 | 1 | 4 |
| Patient 7 germ cell neoplasia in situ | 101 | 71,624,508 | 71,534,605 | 99.87% | 1,756,985 | 2.45 | 91.12 | 4.01 | 2050 | 3611 | 53 | 67 | 1 | 3 |
| Patient 8 germ cell neoplasia in situ | 101 | 97,797,698 | 97,685,716 | 99.89% | 2,595,989 | 2.65 | 90.31 | 4.71 | 2282 | 4004 | 81 | 94 | 2 | 4 |
| Patient 8 seminoma | 101 | 93,810,255 | 93,686,342 | 99.87% | 2,392,233 | 2.55 | 90.58 | 4.66 | 1957 | 3623 | 54 | 81 | 1 | 4 |
| Patient 9 germ cell neoplasia in situ | 101 | 106,946,340 | 106,814,512 | 99.88% | 3,355,668 | 3.14 | 91.20 | 3.63 | 2690 | 4062 | 83 | 104 | 2 | 4 |
| Patient 9 seminoma | 101 | 103,972,264 | 103,841,243 | 99.87% | 3,105,844 | 2.99 | 91.47 | 3.80 | 2077 | 3519 | 66 | 89 | 2 | 4 |
| Patient 10 seminoma | 101 | 81,970,564 | 81,892,350 | 99.90% | 2,492,540 | 3.04 | 89.97 | 4.49 | 2160 | 3752 | 64 | 77 | 1 | 3 |

**Table S3. Summary of chromosomal rearrangements**

| Patient | Histological type | Supporting fragments | Chromosome A | Position A | *Gene A* | Chromosome B | Position B | Gene B | Prediction | Prediction details |
|---|---|---|---|---|---|---|---|---|---|---|
| Patient 1 | Teratoma | 6 | 1 | 78476518 | Non-genic | 11 | 120744717 | *GRIK4* | Truncation | balanced |
| Patient 1 | Teratoma | 10 | 1 | 81478298 | Non-genic | 14 | 22636565 | Non-genic | | |
| Patient 1 | Teratoma | 31 | 2 | 82677850 | Non-genic | 6 | 157645485 | *ZDHHC14* | Truncation | *ZDHHC14*->noGene |
| Patient 1 | Teratoma | 5 | 2 | 178063575 | *PDE11A* | 18 | 54753959 | Non-genic | Truncation | noGene->*PDE11A* |
| Patient 1 | Teratoma | 12 | 2 | 181524121 | *ITGA4* | 6 | 157153664 | *ARID1B* | | |
| Patient 1 | Teratoma | 5 | 3 | 17469347 | *TBC1D5* | 3 | 17600099 | *TBC1D5* | | |
| Patient 1 | Teratoma | 5 | 4 | 177411375 | Non-genic | 8 | 88055270 | *MMP16* | Truncation | noGene->*MMP16* |
| Patient 1 | Teratoma | 7 | 5 | 2455030 | Non-genic | 9 | 99580930 | Non-genic | | |
| Patient 1 | Teratoma | 18 | 5 | 40177273 | Non-genic | 5 | 40975679 | *C7* | Truncation | noGene->*C7* |
| Patient 1 | Teratoma | 19 | 5 | 153525099 | *GRIA1* | 12 | 130059545 | Non-genic | Truncation | balanced |
| Patient 1 | Teratoma | 8 | 6 | 34479252 | *PACSIN1* | 14 | 22636512 | Non-genic | Truncation | *PACSIN1*->noGene |
| Patient 1 | Teratoma | 5 | 6 | 60545662 | Non-genic | 6 | 163410619 | *CAHM* | Truncation | *CAHM*->noGene |
| Patient 1 | Teratoma | 8 | 7 | 14902106 | Non-genic | 23 | 84294816 | Non-genic | | |
| Patient 1 | Teratoma | 6 | 7 | 22770492 | Non-genic | 9 | 76189308 | *PCSK5* | Truncation | noGene->*PCSK5* |
| Patient 1 | Teratoma | 5 | 8 | 136961206 | Non-genic | 11 | 36049486 | *LDLRAD3* | Truncation | balanced |
| Patient 1 | Teratoma | 31 | 10 | 90322654 | Non-genic | 10 | 90846028 | *HTR7* | Truncation | noGene->*HTR7* |
| Patient 1 | Teratoma | 5 | 17 | 70815558 | Non-genic | 17 | 70937825 | Non-genic | | |
| Patient 1 | Teratoma | 6 | 20 | 15145981 | *MACROD2* | 20 | 15263566 | *MACROD2* | | |
| Patient 1 | Yolk sac | 5 | 1 | 81477410 | Non-genic | 14 | 22638573 | Non-genic | | |
| Patient 1 | Yolk sac | 15 | 1 | 145707433 | *PDZK1* | 16 | 83661035 | *CDH13* | Fusion | *PDZK1->CDH13* |
| Patient 1 | Yolk sac | 6 | 2 | 77512665 | *LRRTM4* | 2 | 78062857 | *LOC101927967* | Fusion | *LOC101927967->LRRTM4* |
| Patient 1 | Yolk sac | 24 | 2 | 82677791 | Non-genic | 6 | 157644993 | *ZDHHC14* | Truncation | *ZDHHC14*->noGene |
| Patient 1 | Yolk sac | 16 | 2 | 103810007 | Non-genic | 16 | 83803126 | *LOC102724163* | Truncation | noGene->*LOC102724163* |
| Patient 1 | Yolk sac | 12 | 3 | 4870287 | Non-genic | 3 | 4980640 | *BHLHE40* | Truncation | noGene->*BHLHE40* |
| Patient 1 | Yolk sac | 10 | 3 | 6849175 | Non-genic | 3 | 129163513 | *ISY1* | Truncation | balanced |
| Patient 1 | Yolk sac | 7 | 3 | 6850953 | Non-genic | 3 | 129164026 | *ISY1* | Truncation | *ISY1*->noGene |
| Patient 1 | Yolk sac | 21 | 5 | 40176656 | Non-genic | 5 | 40976301 | *C7* | Truncation | noGene->*C7* |
| Patient 1 | Yolk sac | 8 | 6 | 60545686 | Non-genic | 6 | 163410824 | *CAHM* | Truncation | *CAHM*->noGene |
| Patient 1 | Yolk sac | 14 | 7 | 7361666 | *COL28A1* | 7 | 14778312 | *DGKB* | | |
| Patient 1 | Yolk sac | 10 | 7 | 69627946 | *AUTS2* | 7 | 69788929 | *AUTS2* | | |
| Patient 1 | Yolk sac | 7 | 8 | 58582429 | *SDCBP* | 21 | 37387511 | *DYRK1A* | | |

**Table S3. Summary of chromosomal rearrangements**

| Patient | Histological type | Supporting fragments | Chromosome A | Position A | *Gene A* | Chromosome B | Position B | Gene B | Prediction | Prediction details |
|---|---|---|---|---|---|---|---|---|---|---|
| Patient 1 | Yolk sac | 24 | 10 | 90322910 | Non-genic | 10 | 90846242 | *HTR7* | Truncation | noGene->*HTR7* |
| Patient 1 | Yolk sac | 9 | 11 | 81221692 | Non-genic | 11 | 81352398 | Non-genic | | |
| Patient 1 | Yolk sac | 18 | 11 | 81515892 | Non-genic | 11 | 81667348 | Non-genic | | |
| Patient 1 | Yolk sac | 11 | 14 | 100707163 | Non-genic | 14 | 100962797 | *SNORD114-7* | Truncation | *SNORD114-7*->noGene |
| Patient 1 | Yolk sac | 14 | 18 | 57020349 | *WDR7* | 18 | 57551699 | *FECH* | | |
| Patient 1 | Yolk sac | 20 | 21 | 23037321 | Non-genic | 21 | 23224724 | Non-genic | | |
| Patient 2 | Teratoma | 6 | 1 | 61938637 | *PATJ* | 18 | 34735208 | *DTNA* | Fusion | *PATJ->DTNA* |
| Patient 2 | Teratoma | 6 | 1 | 171214499 | *FMO2* | 23 | 132979255 | Non-genic | Truncation | *FMO2*->noGene |
| Patient 2 | Teratoma | 7 | 1 | 185638465 | Non-genic | 3 | 86980715 | *VGLL3* | Truncation | noGene->*VGLL3* |
| Patient 2 | Teratoma | 73 | 2 | 159588 | Non-genic | 22 | 25190719 | *KIAA1671* | Truncation | *KIAA1671*->noGene |
| Patient 2 | Teratoma | 15 | 2 | 164829 | Non-genic | 22 | 25190650 | *KIAA1671* | Truncation | *KIAA1671*->noGene |
| Patient 2 | Teratoma | 40 | 2 | 26404231 | *DRC1* | 16 | 68806453 | *CDH1* | | |
| Patient 2 | Teratoma | 5 | 2 | 111067041 | *ACOXL* | 7 | 129691412 | *NRF1* | | |
| Patient 2 | Teratoma | 5 | 2 | 217226447 | Non-genic | 10 | 98763282 | *HPSE2* | Truncation | *HPSE2*->noGene |
| Patient 2 | Teratoma | 5 | 2 | 236971326 | Non-genic | 6 | 34108262 | *GRM4* | Truncation | *GRM4*->noGene |
| Patient 2 | Teratoma | 5 | 3 | 136359819 | *STAG1* | 5 | 173367519 | Non-genic | Truncation | *STAG1*->noGene |
| Patient 2 | Teratoma | 6 | 3 | 147012375 | Non-genic | 7 | 68293224 | Non-genic | | |
| Patient 2 | Teratoma | 15 | 4 | 19238328 | Non-genic | 4 | 19356736 | Non-genic | | |
| Patient 2 | Teratoma | 14 | 4 | 127823692 | *HSPA4L* | 4 | 128339653 | Non-genic | Truncation | noGene->*HSPA4L* |
| Patient 2 | Teratoma | 5 | 8 | 24790771 | Non-genic | 23 | 8808261 | Non-genic | | |
| Patient 2 | Teratoma | 5 | 8 | 43458409 | Non-genic | 14 | 89836122 | *EFCAB11* | Truncation | *EFCAB11*->noGene |
| Patient 2 | Teratoma | 13 | 8 | 47022844 | Non-genic | 8 | 47154916 | Non-genic | | |
| Patient 2 | Teratoma | 12 | 9 | 32527786 | *DDX58* | 17 | 73486107 | *SDK2* | | |
| Patient 2 | Teratoma | 6 | 10 | 34866278 | Non-genic | 11 | 80055257 | Non-genic | | |
| Patient 2 | Teratoma | 5 | 11 | 88183164 | Non-genic | 23 | 47289181 | Non-genic | | |
| Patient 2 | Teratoma | 11 | 12 | 56629044 | *BAZ2A* | 12 | 56789203 | *HSD17B6* | | |
| Patient 2 | Teratoma | 6 | 13 | 27623164 | *POLR1D* | 21 | 41665116 | Non-genic | Truncation | *POLR1D*->noGene |
| Patient 2 | Teratoma | 62 | 15 | 70557630 | Non-genic | 15 | 70875272 | *LRRC49* | Truncation | *LRRC49*->noGene |
| Patient 2 | Yolk sac | 47 | 2 | 159645 | Non-genic | 22 | 25190761 | *KIAA1671* | Truncation | *KIAA1671*->noGene |
| Patient 2 | Yolk sac | 7 | 2 | 26402966 | *DRC1* | 16 | 68806029 | *CDH1* | | |
| Patient 2 | Yolk sac | 15 | 2 | 26403739 | *DRC1* | 16 | 68806404 | *CDH1* | | |

**Table S3. Summary of chromosomal rearrangements**

| Patient | Histological type | Supporting fragments | Chromosome A | Position A | *Gene A* | Chromosome B | Position B | Gene B | Prediction | Prediction details |
|---|---|---|---|---|---|---|---|---|---|---|
| Patient 2 | Yolk sac | 8 | 4 | 19238472 | Non-genic | 4 | 19356652 | Non-genic | | |
| Patient 2 | Yolk sac | 9 | 4 | 156530811 | Non-genic | 13 | 59201614 | Non-genic | | |
| Patient 2 | Yolk sac | 9 | 8 | 47022782 | Non-genic | 8 | 47154483 | Non-genic | | |
| Patient 3 | Teratoma | 57 | 1 | 41108726 | *SCMH1* | 1 | 41370561 | *FOXO6* | | |
| Patient 3 | Teratoma | 18 | 1 | 45483972 | *TESK2* | 1 | 45841341 | *MAST2* | | |
| Patient 3 | Teratoma | 5 | 2 | 21168968 | Non-genic | 23 | 11713285 | Non-genic | | |
| Patient 3 | Teratoma | 20 | 2 | 206670767 | *DYTN* | 2 | 207909074 | *PLEKHM3* | Fusion | *DYTN->PLEKHM3* |
| Patient 3 | Teratoma | 36 | 3 | 27104535 | Non-genic | 3 | 27448685 | *SLC4A7* | Truncation | *SLC4A7*->noGene |
| Patient 3 | Teratoma | 8 | 3 | 48763100 | *PRKAR2A* | 3 | 48949578 | *ARIH2* | | |
| Patient 3 | Teratoma | 16 | 3 | 193613390 | *OPA1* | 3 | 193839316 | Non-genic | Truncation | noGene->*OPA1* |
| Patient 3 | Teratoma | 22 | 4 | 74249036 | *MTHFD2L* | 5 | 39347413 | *C9* | Fusion | balanced |
| Patient 3 | Teratoma | 10 | 7 | 20726943 | *ABCB5* | 7 | 20875243 | *LINC01162* | Fusion | *LINC01162->ABCB5* |
| Patient 3 | Teratoma | 8 | 9 | 33993330 | *UBAP2* | 9 | 34235126 | *UBAP1* | | |
| Patient 3 | Teratoma | 5 | 10 | 20921008 | *NEBL* | 10 | 25320124 | *GPR158* | Fusion | *NEBL->GPR158* |
| Patient 3 | Teratoma | 5 | 10 | 70019547 | Non-genic | 10 | 70202598 | *PPA1* | Truncation | noGene->*PPA1* |
| Patient 3 | Teratoma | 10 | 11 | 65159610 | Non-genic | 11 | 65324871 | *CDC42EP2* | Truncation | *CDC42EP2*->noGene |
| Patient 3 | Teratoma | 10 | 11 | 65437196 | *NEAT1* | 11 | 65654937 | *RELA* | | |
| Patient 3 | Teratoma | 7 | 12 | 73774807 | Non-genic | 12 | 73975336 | Non-genic | | |
| Patient 3 | Teratoma | 39 | 14 | 78795625 | *NRXN3* | 14 | 78961679 | *NRXN3* | | |
| Patient 3 | Teratoma | 22 | 17 | 46189191 | *KANSL1* | 17 | 46450428 | Non-genic | Truncation | *KANSL1*->noGene |
| Patient 3 | Teratoma | 17 | 19 | 19626332 | *LPAR2* | 19 | 20627061 | *ZNF626* | Fusion | *LPAR2->ZNF626* |
| Patient 3 | Teratoma | 11 | 24 | 8176721 | Non-genic | 24 | 9265297 | Non-genic | | |
| Patient 4 | Embryonal carcinoma | 5 | 1 | 18687768 | *PAX7* | 5 | 162126877 | *GABRG2* | Fusion | *PAX7->GABRG2* |
| Patient 4 | Embryonal carcinoma | 9 | 1 | 21242803 | *ECE1* | 12 | 84675064 | Non-genic | Truncation | balanced |
| Patient 4 | Embryonal carcinoma | 7 | 1 | 46905300 | Non-genic | 3 | 115126720 | *ZBTB20* | Truncation | balanced |
| Patient 4 | Embryonal carcinoma | 5 | 1 | 60174668 | Non-genic | 12 | 14076962 | Non-genic | | |
| Patient 4 | Embryonal carcinoma | 7 | 1 | 228827644 | Non-genic | 16 | 12179460 | *SNX29* | Truncation | *SNX29*->noGene |
| Patient 4 | Embryonal carcinoma | 6 | 1 | 230411621 | *PGBD5* | 7 | 128563593 | Non-genic | Truncation | balanced |
| Patient 4 | Embryonal carcinoma | 5 | 2 | 177798772 | *PDE11A* | 3 | 175811550 | Non-genic | Truncation | balanced |
| Patient 4 | Embryonal carcinoma | 5 | 2 | 231794981 | *COPS7B* | 23 | 121299209 | Non-genic | Truncation | balanced |
| Patient 4 | Embryonal carcinoma | 44 | 3 | 38903 | Non-genic | 3 | 139956234 | *CLSTN2* | Truncation | noGene->*CLSTN2* |

**Table S3. Summary of chromosomal rearrangements**

| Patient | Histological type | Supporting fragments | Chromosome A | Position A | *Gene A* | Chromosome B | Position B | Gene B | Prediction | Prediction details |
|---|---|---|---|---|---|---|---|---|---|---|
| Patient 4 | Embryonal carcinoma | 5 | 3 | 57443512 | *DNAH12* | 17 | 80857258 | *RPTOR* | Fusion | balanced |
| Patient 4 | Embryonal carcinoma | 5 | 3 | 73701123 | Non-genic | 13 | 73794521 | *KLF12* | Truncation | *KLF12*->noGene |
| Patient 4 | Embryonal carcinoma | 38 | 3 | 100616382 | *ADGRG7* | 3 | 100727176 | *TFG* | Fusion | *TFG->ADGRG7* |
| Patient 4 | Embryonal carcinoma | 15 | 3 | 108434052 | *MYH15* | 6 | 35838933 | *SRPK1* | Fusion | balanced |
| Patient 4 | Embryonal carcinoma | 10 | 3 | 130593056 | *COL6A6* | 5 | 43553990 | *PAIP1* | | |
| Patient 4 | Embryonal carcinoma | 7 | 4 | 60399279 | Non-genic | 8 | 4087292 | *CSMD1* | Truncation | noGene->*CSMD1* |
| Patient 4 | Embryonal carcinoma | 6 | 5 | 14113018 | Non-genic | 22 | 41500647 | *ACO2* | Truncation | noGene->*ACO2* |
| Patient 4 | Embryonal carcinoma | 6 | 5 | 22690255 | *CDH12* | 11 | 16002992 | *SOX6* | | |
| Patient 4 | Embryonal carcinoma | 58 | 5 | 73840094 | *ARHGEF28* | 14 | 77769529 | *C14orf178* | Fusion | balanced |
| Patient 4 | Embryonal carcinoma | 7 | 5 | 98871548 | *CHD1* | 7 | 73922446 | Non-genic | Truncation | balanced |
| Patient 4 | Embryonal carcinoma | 5 | 5 | 166306255 | Non-genic | 14 | 53518070 | Non-genic | | |
| Patient 4 | Embryonal carcinoma | 5 | 5 | 175426902 | Non-genic | 15 | 80099539 | *ZFAND6* | Truncation | *ZFAND6*->noGene |
| Patient 4 | Embryonal carcinoma | 5 | 6 | 115880498 | Non-genic | 16 | 59605748 | Non-genic | | |
| Patient 4 | Embryonal carcinoma | 10 | 6 | 157403702 | *ZDHHC14* | 8 | 80138896 | *TPD52* | Fusion | balanced |
| Patient 4 | Embryonal carcinoma | 6 | 7 | 108365015 | *NRCAM* | 15 | 37909564 | Non-genic | Truncation | balanced |
| Patient 4 | Embryonal carcinoma | 9 | 8 | 10749215 | Non-genic | 11 | 131948463 | *NTM* | Truncation | *NTM*->noGene |
| Patient 4 | Embryonal carcinoma | 5 | 8 | 21318132 | Non-genic | 8 | 21429853 | Non-genic | | |
| Patient 4 | Embryonal carcinoma | 5 | 8 | 57518515 | Non-genic | 15 | 92179358 | Non-genic | | |
| Patient 4 | Embryonal carcinoma | 5 | 8 | 128282266 | Non-genic | 15 | 34700452 | Non-genic | | |
| Patient 4 | Embryonal carcinoma | 6 | 8 | 132395763 | *KCNQ3* | 14 | 76657535 | Non-genic | Truncation | balanced |
| Patient 4 | Embryonal carcinoma | 8 | 10 | 79935599 | *SFTPD* | 13 | 57555493 | Non-genic | Truncation | balanced |
| Patient 4 | Embryonal carcinoma | 6 | 12 | 75819781 | Non-genic | 14 | 48138483 | Non-genic | | |
| Patient 4 | Embryonal carcinoma | 41 | 12 | 96971662 | Non-genic | 13 | 60179785 | Non-genic | | |
| Patient 4 | Embryonal carcinoma | 6 | 13 | 29843054 | *UBL3* | 17 | 34675677 | Non-genic | Truncation | balanced |
| Patient 4 | Embryonal carcinoma | 6 | 13 | 44409012 | *TUSC8* | 23 | 84907863 | Non-genic | Truncation | noGene->*TUSC8* |
| Patient 4 | Embryonal carcinoma | 5 | 13 | 66591342 | *PCDH9* | 22 | 48436354 | Non-genic | Truncation | balanced |
| Patient 4 | Embryonal carcinoma | 40 | 18 | 5155352 | *AKAIN1* | 18 | 5308387 | Non-genic | Truncation | balanced |
| Patient 4 | Germ cell neoplasia in situ | 67 | 3 | 100615999 | *ADGRG7* | 3 | 100727224 | *TFG* | Fusion | *TFG->ADGRG7* |
| Patient 4 | Germ cell neoplasia in situ | 6 | 11 | 93628436 | Non-genic | 11 | 94090300 | *HEPHL1* | Truncation | balanced |
| Patient 4 | Germ cell neoplasia in situ | 35 | 12 | 96971636 | Non-genic | 13 | 60172659 | Non-genic | | |
| Patient 4 | Germ cell neoplasia in situ | 5 | 14 | 20427485 | *KLHL33* | 19 | 29900016 | Non-genic | Truncation | balanced |

**Table S3. Summary of chromosomal rearrangements**

| Patient | Histological type | Supporting fragments | Chromosome A | Position A | *Gene A* | Chromosome B | Position B | Gene B | Prediction | Prediction details |
|---|---|---|---|---|---|---|---|---|---|---|
| Patient 4 | Normal | 74 | 3 | 100616078 | *ADGRG7* | 3 | 100727212 | *TFG* | Fusion | *TFG->ADGRG7* |
| Patient 4 | Normal | 6 | 5 | 8278640 | Non-genic | 13 | 92557420 | *GPC5* | Truncation | balanced |
| Patient 4 | Normal | 27 | 12 | 96969612 | Non-genic | 13 | 60179862 | Non-genic | | |
| Patient 4 | Teratoma | 5 | 1 | 118598975 | Non-genic | 12 | 42962689 | Non-genic | | |
| Patient 4 | Teratoma | 5 | 2 | 160177583 | *ITGB6* | 11 | 17822857 | *SERGEF* | | |
| Patient 4 | Teratoma | 53 | 3 | 38927 | Non-genic | 3 | 139956206 | *CLSTN2* | Truncation | noGene->*CLSTN2* |
| Patient 4 | Teratoma | 6 | 3 | 73548649 | *PDZRN3* | 17 | 19441016 | Non-genic | Truncation | *PDZRN3*->noGene |
| Patient 4 | Teratoma | 67 | 3 | 100616035 | *ADGRG7* | 3 | 100727245 | *TFG* | Fusion | *TFG->ADGRG7* |
| Patient 4 | Teratoma | 10 | 3 | 130961218 | *ATP2C1* | 9 | 40926558 | *MIR1299* | Fusion | *MIR1299->ATP2C1* |
| Patient 4 | Teratoma | 5 | 3 | 165091772 | Non-genic | 14 | 100909598 | *MIR370* | Truncation | noGene->*MIR370* |
| Patient 4 | Teratoma | 5 | 3 | 172685539 | *NCEH1* | 3 | 195137007 | *XXYLT1* | Fusion | *NCEH1->XXYLT1* |
| Patient 4 | Teratoma | 7 | 3 | 173645499 | *NLGN1* | 3 | 175325082 | *NAALADL2* | Fusion | *NLGN1->NAALADL2* |
| Patient 4 | Teratoma | 64 | 5 | 73835122 | *ARHGEF28* | 14 | 77769419 | *C14orf178* | Fusion | *ARHGEF28->C14orf178* |
| Patient 4 | Teratoma | 36 | 5 | 73835621 | *ARHGEF28* | 14 | 77778869 | Non-genic | Truncation | noGene->*ARHGEF28* |
| Patient 4 | Teratoma | 6 | 7 | 146691835 | *CNTNAP2* | 7 | 146791853 | *CNTNAP2* | | |
| Patient 4 | Teratoma | 6 | 9 | 135943961 | *UBAC1* | 18 | 59821230 | Non-genic | Truncation | noGene->*UBAC1* |
| Patient 4 | Teratoma | 8 | 11 | 59632782 | Non-genic | 23 | 22286968 | *PTCHD1-AS* | Truncation | *PTCHD1-AS*->noGene |
| Patient 4 | Teratoma | 5 | 12 | 571906 | *NINJ2* | 12 | 32130351 | *BICD1* | | |
| Patient 4 | Teratoma | 59 | 12 | 3236339 | *TSPAN9* | 12 | 3341672 | Non-genic | Truncation | *TSPAN9*->noGene |
| Patient 4 | Teratoma | 6 | 12 | 31503346 | *DENND5B* | 12 | 31623315 | Non-genic | Truncation | *DENND5B*->noGene |
| Patient 4 | Teratoma | 50 | 12 | 96969135 | Non-genic | 13 | 60185985 | Non-genic | | |
| Patient 4 | Teratoma | 17 | 16 | 78978832 | *WWOX* | 16 | 79122769 | *WWOX* | | |
| Patient 4 | Teratoma | 26 | 23 | 97059471 | *DIAPH2* | 23 | 97648319 | Non-genic | Truncation | noGene->*DIAPH2* |
| Patient 5 | Embryonal carcinoma | 5 | 2 | 205825740 | Non-genic | 8 | 37352262 | Non-genic | | |
| Patient 5 | Embryonal carcinoma | 9 | 3 | 196501463 | *RNF168* | 3 | 196733943 | *PIGX* | | |
| Patient 5 | Embryonal carcinoma | 6 | 4 | 34562215 | Non-genic | 18 | 80241563 | *PARD6G* | Truncation | *PARD6G*->noGene |
| Patient 5 | Embryonal carcinoma | 8 | 4 | 61043889 | Non-genic | 8 | 55747208 | *TMEM68* | Truncation | noGene->*TMEM68* |
| Patient 5 | Embryonal carcinoma | 21 | 4 | 62988790 | Non-genic | 4 | 63328199 | Non-genic | | |
| Patient 5 | Embryonal carcinoma | 30 | 4 | 163804763 | *MARCH1* | 7 | 115076946 | *LINC01393* | | |
| Patient 5 | Embryonal carcinoma | 13 | 5 | 155129159 | Non-genic | 5 | 156534942 | *SGCD* | Truncation | balanced |
| Patient 5 | Embryonal carcinoma | 9 | 6 | 44910260 | *SUPT3H* | 8 | 55814340 | *TGS1* | | |

**Table S3. Summary of chromosomal rearrangements**

| Patient | Histological type | Supporting fragments | Chromosome A | Position A | *Gene A* | Chromosome B | Position B | Gene B | Prediction | Prediction details |
|---|---|---|---|---|---|---|---|---|---|---|
| Patient 5 | Embryonal carcinoma | 41 | 6 | 142949424 | Non-genic | 6 | 143178261 | *AIG1* | Truncation | *AIG1*->noGene |
| Patient 5 | Embryonal carcinoma | 61 | 7 | 130570059 | *COPG2* | 10 | 117672695 | Non-genic | Truncation | noGene->*COPG2* |
| Patient 5 | Embryonal carcinoma | 31 | 8 | 53624595 | Non-genic | 17 | 10978198 | Non-genic | | |
| Patient 5 | Embryonal carcinoma | 5 | 9 | 124807973 | *OLFML2A* | 9 | 124972059 | *SCAI* | | |
| Patient 5 | Embryonal carcinoma | 20 | 10 | 125619971 | *TEX36* | 12 | 69080617 | Non-genic | Truncation | *TEX36*->noGene |
| Patient 5 | Embryonal carcinoma | 66 | 10 | 132999561 | Non-genic | 12 | 107112473 | Non-genic | | |
| Patient 5 | Embryonal carcinoma | 6 | 16 | 236278 | *FAM234A* | 20 | 62161957 | *SS18L1* | Fusion | *SS18L1->FAM234A* |
| Patient 5 | Embryonal carcinoma | 44 | 16 | 7357463 | *RBFOX1* | 16 | 7572633 | *RBFOX1* | | |
| Patient 5 | Germ cell neoplasia in situ | 6 | 1 | 109308938 | *SORT1* | 22 | 26649117 | Non-genic | Truncation | *SORT1*->noGene |
| Patient 5 | Germ cell neoplasia in situ | 5 | 4 | 61043480 | Non-genic | 8 | 55747282 | *TMEM68* | Truncation | noGene->*TMEM68* |
| Patient 5 | Germ cell neoplasia in situ | 32 | 4 | 62988523 | Non-genic | 4 | 63328646 | Non-genic | | |
| Patient 5 | Germ cell neoplasia in situ | 8 | 4 | 163804803 | *MARCH1* | 7 | 115075982 | *LINC01393* | | |
| Patient 5 | Germ cell neoplasia in situ | 33 | 6 | 142949643 | Non-genic | 6 | 143178231 | *AIG1* | Truncation | *AIG1*->noGene |
| Patient 5 | Germ cell neoplasia in situ | 11 | 7 | 56824422 | Non-genic | 7 | 57436715 | Non-genic | | |
| Patient 5 | Germ cell neoplasia in situ | 8 | 8 | 53624619 | Non-genic | 17 | 10978292 | Non-genic | | |
| Patient 5 | Germ cell neoplasia in situ | 7 | 10 | 132998237 | Non-genic | 12 | 107112024 | Non-genic | | |
| Patient 5 | Normal | 10 | 3 | 34176644 | Non-genic | 11 | 108975323 | Non-genic | | |
| Patient 5 | Normal | 42 | 4 | 62988591 | Non-genic | 4 | 63329196 | Non-genic | | |
| Patient 5 | Normal | 37 | 6 | 142949364 | Non-genic | 6 | 143178231 | *AIG1* | Truncation | *AIG1*->noGene |
| Patient 5 | Normal | 6 | 7 | 56824049 | Non-genic | 7 | 57436440 | Non-genic | | |
| Patient 5 | Normal | 5 | 9 | 97437187 | *TDRD7* | 15 | 40584668 | Non-genic | Truncation | balanced |
| Patient 5 | Normal | 6 | 12 | 19518397 | *AEBP2* | 12 | 130374796 | *PIWIL1* | | |
| Patient 6 | Embryonal carcinoma | 5 | 1 | 16013944 | *HSPB7* | 3 | 131982974 | *CPNE4* | | |
| Patient 6 | Embryonal carcinoma | 14 | 1 | 118856094 | Non-genic | 8 | 120773873 | *SNTB1* | Truncation | balanced |
| Patient 6 | Embryonal carcinoma | 13 | 2 | 723748 | Non-genic | 20 | 56696616 | Non-genic | | |
| Patient 6 | Embryonal carcinoma | 5 | 2 | 27254489 | *SLC30A3* | 7 | 100589554 | *PCOLCE-AS1* | Fusion | *SLC30A3->PCOLCE-AS1* |
| Patient 6 | Embryonal carcinoma | 10 | 2 | 50003844 | *NRXN1* | 2 | 50562986 | *NRXN1* | | |
| Patient 6 | Embryonal carcinoma | 6 | 2 | 123867835 | Non-genic | 2 | 126546913 | Non-genic | | |
| Patient 6 | Embryonal carcinoma | 6 | 4 | 75042673 | *PARM1* | 5 | 128567315 | Non-genic | Truncation | *PARM1*->noGene |
| Patient 6 | Embryonal carcinoma | 13 | 4 | 100188043 | *DDIT4L* | 4 | 100306986 | Non-genic | Truncation | noGene->*DDIT4L* |
| Patient 6 | Embryonal carcinoma | 13 | 5 | 13178 | Non-genic | 5 | 238811 | *SDHA* | Truncation | *SDHA*->noGene |

**Table S3. Summary of chromosomal rearrangements**

| Patient | Histological type | Supporting fragments | Chromosome A | Position A | *Gene A* | Chromosome B | Position B | Gene B | Prediction | Prediction details |
|---|---|---|---|---|---|---|---|---|---|---|
| Patient 6 | Embryonal carcinoma | 7 | 5 | 42777262 | *CCDC152* | 6 | 102395714 | Non-genic | Truncation | noGene->*CCDC152* |
| Patient 6 | Embryonal carcinoma | 68 | 5 | 115887184 | *AP3S1* | 5 | 116115943 | *COMMD10* | Fusion | *COMMD10->AP3S1* |
| Patient 6 | Embryonal carcinoma | 6 | 6 | 2004147 | *GMDS* | 19 | 36168644 | Non-genic | Truncation | *GMDS*->noGene |
| Patient 6 | Embryonal carcinoma | 5 | 6 | 19996833 | Non-genic | 6 | 32071595 | *TNXB* | Truncation | *TNXB*->noGene |
| Patient 6 | Embryonal carcinoma | 16 | 6 | 156434107 | Non-genic | 12 | 27511738 | Non-genic | | |
| Patient 6 | Embryonal carcinoma | 18 | 6 | 161596552 | *PARK2* | 12 | 31028865 | *DDX11-AS1* | | |
| Patient 6 | Embryonal carcinoma | 5 | 8 | 30242613 | Non-genic | 9 | 122814547 | Non-genic | | |
| Patient 6 | Embryonal carcinoma | 7 | 9 | 21811394 | *MTAP* | 12 | 48670555 | *KANSL2* | | |
| Patient 6 | Embryonal carcinoma | 7 | 12 | 8536139 | *CLEC4E* | 12 | 12083724 | *BCL2L14* | Fusion | *BCL2L14->CLEC4E* |
| Patient 6 | Embryonal carcinoma | 58 | 23 | 155326592 | *CLIC2* | 23 | 155440982 | *Non-genic* | Truncation | *CLIC2*->noGene |
| Patient 6 | Normal | 13 | 1 | 65141854 | Non-genic | 23 | 18066108 | *LINC01456* | Truncation | balanced |
| Patient 6 | Normal | 15 | 1 | 118858427 | Non-genic | 8 | 120775285 | *SNTB1* | Truncation | balanced |
| Patient 6 | Normal | 24 | 5 | 13325 | Non-genic | 5 | 238778 | *SDHA* | Truncation | *SDHA*->noGene |
| Patient 6 | Normal | 9 | 5 | 42777556 | *CCDC152* | 6 | 102395705 | Non-genic | Truncation | noGene->*CCDC152* |
| Patient 6 | Normal | 49 | 5 | 115887423 | *AP3S1* | 5 | 116115887 | *COMMD10* | Fusion | *COMMD10->AP3S1* |
| Patient 6 | Normal | 53 | 23 | 155326543 | *CLIC2* | 23 | 155441006 | Non-genic | Truncation | *CLIC2*->noGene |
| Patient 7 | Embryonal carcinoma | 6 | 1 | 213264051 | *RPS6KC1* | 6 | 51631310 | *PKHD1* | Fusion | balanced |
| Patient 7 | Embryonal carcinoma | 5 | 2 | 78632396 | Non-genic | 18 | 22473519 | Non-genic | | |
| Patient 7 | Embryonal carcinoma | 39 | 3 | 30315 | Non-genic | 3 | 146408206 | Non-genic | | |
| Patient 7 | Embryonal carcinoma | 17 | 3 | 42141 | Non-genic | 3 | 146408553 | Non-genic | | |
| Patient 7 | Embryonal carcinoma | 5 | 4 | 7081264 | Non-genic | 12 | 22952315 | Non-genic | | |
| Patient 7 | Embryonal carcinoma | 27 | 4 | 47145121 | *GABRB1* | 4 | 47252242 | *GABRB1* | | |
| Patient 7 | Embryonal carcinoma | 6 | 5 | 37954400 | Non-genic | 12 | 6327175 | *PLEKHG6* | Truncation | *PLEKHG6*->noGene |
| Patient 7 | Embryonal carcinoma | 7 | 5 | 39461075 | Non-genic | 21 | 21777908 | *LINC01425* | Truncation | balanced |
| Patient 7 | Embryonal carcinoma | 5 | 5 | 54098510 | *ARL15* | 5 | 134531431 | *JADE2* | | |
| Patient 7 | Embryonal carcinoma | 9 | 5 | 59028340 | *PDE4D* | 7 | 1437892 | *MICALL2* | Fusion | balanced |
| Patient 7 | Embryonal carcinoma | 5 | 5 | 99767695 | Non-genic | 7 | 113286032 | Non-genic | | |
| Patient 7 | Embryonal carcinoma | 5 | 6 | 96229818 | Non-genic | 23 | 29140766 | *IL1RAPL1* | Truncation | balanced |
| Patient 7 | Embryonal carcinoma | 19 | 6 | 133627823 | *TARID* | 6 | 134334116 | Non-genic | Truncation | *TARID*->noGene |
| Patient 7 | Embryonal carcinoma | 23 | 6 | 162162565 | *PARK2* | 6 | 162667092 | *PARK2* | | |
| Patient 7 | Embryonal carcinoma | 9 | 7 | 32302386 | *PDE1C* | 15 | 88456779 | *MRPL46* | | |

**Table S3. Summary of chromosomal rearrangements**

| Patient | Histological type | Supporting fragments | Chromosome A | Position A | *Gene A* | Chromosome B | Position B | Gene B | Prediction | Prediction details |
|---|---|---|---|---|---|---|---|---|---|---|
| Patient 7 | Embryonal carcinoma | 5 | 7 | 50507735 | *DDC* | 23 | 150929648 | Non-genic | Truncation | *DDC*->noGene |
| Patient 7 | Embryonal carcinoma | 5 | 9 | 16762947 | *BNC2* | 16 | 85229070 | Non-genic | Truncation | noGene->*BNC2* |
| Patient 7 | Embryonal carcinoma | 39 | 10 | 312159 | *DIP2C* | 10 | 581425 | *DIP2C* | | |
| Patient 7 | Embryonal carcinoma | 8 | 10 | 118971890 | Non-genic | 15 | 38707196 | Non-genic | | |
| Patient 7 | Embryonal carcinoma | 5 | 12 | 24341199 | *SOX5* | 23 | 101593846 | Non-genic | Truncation | *SOX5*->noGene |
| Patient 7 | Embryonal carcinoma | 5 | 13 | 35562690 | *NBEA* | 15 | 20287881 | *CHEK2P2* | Fusion | *NBEA->CHEK2P2* |
| Patient 7 | Embryonal carcinoma | 27 | 16 | 5372683 | Non-genic | 16 | 5534858 | Non-genic | | |
| Patient 7 | Germ cell neoplasia in situ | 7 | 2 | 205368097 | *PARD3B* | 20 | 56933508 | Non-genic | Truncation | noGene->*PARD3B* |
| Patient 7 | Germ cell neoplasia in situ | 6 | 3 | 63221742 | Non-genic | 5 | 159592689 | Non-genic | | |
| Patient 7 | Germ cell neoplasia in situ | 6 | 6 | 98533262 | Non-genic | 14 | 54292829 | Non-genic | | |
| Patient 7 | Germ cell neoplasia in situ | 18 | 16 | 5372691 | Non-genic | 16 | 5535182 | Non-genic | | |
| Patient 8 | Germ cell neoplasia in situ | 5 | 1 | 109487771 | *ATXN7L2* | 1 | 111791802 | *KCND3* | | |
| Patient 8 | Germ cell neoplasia in situ | 83 | 2 | 44209555 | *PPM1B* | 2 | 44318557 | *PREPL* | | |
| Patient 8 | Germ cell neoplasia in situ | 7 | 10 | 45997400 | *TIMM23* | 10 | 78999524 | *ZMIZ1-AS1* | Fusion | *ZMIZ1-AS1->TIMM23* |
| Patient 8 | Germ cell neoplasia in situ | 7 | 20 | 15717349 | *MACROD2* | 20 | 15973177 | *LOC613266* | Fusion | *MACROD2->LOC613266* |
| Patient 8 | Seminoma | 16 | 1 | 109487862 | *ATXN7L2* | 1 | 111791423 | *KCND3* | | |
| Patient 8 | Seminoma | 37 | 2 | 44209386 | *PPM1B* | 2 | 44318884 | *PREPL* | | |
| Patient 8 | Seminoma | 21 | 2 | 59237255 | *LINC01793* | 2 | 232467305 | Non-genic | Truncation | *LINC01793*->noGene |
| Patient 8 | Seminoma | 47 | 2 | 198148876 | *PLCL1* | 2 | 198631085 | Non-genic | Truncation | *PLCL1*->noGene |
| Patient 8 | Seminoma | 10 | 6 | 119626430 | *LOC105377975* | 12 | 27699191 | *REP15* | Fusion | *REP15->LOC105377975* |
| Patient 8 | Seminoma | 18 | 10 | 44659108 | Non-genic | 10 | 129639881 | *MGMT* | Truncation | *MGMT*->noGene |
| Patient 8 | Seminoma | 57 | 10 | 45995652 | *TIMM23* | 10 | 78998702 | *ZMIZ1-AS1* | Fusion | *ZMIZ1-AS1->TIMM23* |
| Patient 8 | Seminoma | 6 | 10 | 54470197 | *PCDH15* | 10 | 86991400 | *AGAP11* | | |
| Patient 8 | Seminoma | 18 | 10 | 57402041 | Non-genic | 10 | 96315008 | *DNTT* | Truncation | noGene->*DNTT* |
| Patient 8 | Seminoma | 6 | 10 | 83340409 | Non-genic | 10 | 83699824 | Non-genic | | |
| Patient 8 | Seminoma | 12 | 13 | 19483221 | *TPTE2* | 13 | 21824710 | Non-genic | Truncation | noGene->*TPTE2* |
| Patient 8 | Seminoma | 10 | 13 | 24122451 | *SPATA13* | 19 | 33609412 | Non-genic | Truncation | *SPATA13*->noGene |
| Patient 8 | Seminoma | 8 | 13 | 81956468 | Non-genic | 13 | 82096256 | Non-genic | | |
| Patient 8 | Seminoma | 11 | 20 | 40789221 | Non-genic | 20 | 41275263 | *ZHX3* | Truncation | noGene->*ZHX3* |
| Patient 9 | Germ cell neoplasia in situ | 5 | 1 | 202791426 | *KDM5B* | 6 | 56894976 | *DST* | Fusion | balanced |
| Patient 9 | Germ cell neoplasia in situ | 20 | 4 | 65601786 | *EPHA5* | 15 | 73394540 | Non-genic | Truncation | *EPHA5*->noGene |

**Table S3. Summary of chromosomal rearrangements**

| Patient | Histological type | Supporting fragments | Chromosome A | Position A | *Gene A* | Chromosome B | Position B | Gene B | Prediction | Prediction details |
|---|---|---|---|---|---|---|---|---|---|---|
| Patient 9 | Germ cell neoplasia in situ | 6 | 4 | 159229580 | Non-genic | 7 | 22622843 | Non-genic | | |
| Patient 9 | Germ cell neoplasia in situ | 41 | 5 | 19152140 | Non-genic | 5 | 19394008 | Non-genic | | |
| Patient 9 | Germ cell neoplasia in situ | 16 | 7 | 91290876 | Non-genic | 8 | 136831069 | Non-genic | | |
| Patient 9 | Germ cell neoplasia in situ | 7 | 10 | 68048982 | *HERC4* | 20 | 19868243 | Non-genic | Truncation | *HERC4*->noGene |
| Patient 9 | Seminoma | 31 | 1 | 609540 | Non-genic | 4 | 19583725 | Non-genic | | |
| Patient 9 | Seminoma | 6 | 1 | 37721325 | *EPHA10* | 1 | 99014737 | *LOC100129620* | | |
| Patient 9 | Seminoma | 10 | 4 | 35326171 | Non-genic | 4 | 40641748 | Non-genic | | |
| Patient 9 | Seminoma | 22 | 5 | 19152038 | Non-genic | 5 | 19393894 | Non-genic | | |
| Patient 9 | Seminoma | 6 | 6 | 134597477 | Non-genic | 7 | 116399537 | Non-genic | | |
| Patient 9 | Seminoma | 25 | 12 | 30590811 | Non-genic | 12 | 32957479 | Non-genic | | |
| Patient 10 | Seminoma | 14 | 1 | 72433622 | Non-genic | 1 | 72543889 | Non-genic | | |
| Patient 10 | Seminoma | 38 | 5 | 4687617 | Non-genic | 18 | 74158681 | *TIMM21* | Truncation | balanced |
| Patient 10 | Seminoma | 5 | 20 | 31188995 | Non-genic | 20 | 31777096 | *TPX2* | Truncation | *TPX2*->noGene |

**Table S4. Summary of insertion/deletions and single nucleotide variants**

| Chromosome | Position | Reference allele | Alternate allele | dbSNP | Type of mutation | Gene | Mutation | Normal alternate allele count | Normal total allele count | Tumor alternate allele count | Tumor total allele count | Patient | Histological type |
|---|---|---|---|---|---|---|---|---|---|---|---|---|---|
| 6 | 32581580 | G | A | rs200809587 | Substitution | HLA-DRB1 | c.629C>T_p.Thr210Ile | 0 | 57 | 7 | 66 | 4 | Germ cell neoplasia in situ |
| 6 | 32581748 | C | G | . | Substitution | HLA-DRB1 | c.461G>C_p.Gly154Ala | 0 | 150 | 18 | 198 | 4 | Germ cell neoplasia in situ |
| 6 | 32581754 | T | C | rs112796209 | Substitution | HLA-DRB1 | c.455A>G_p.Tyr152Cys | 0 | 159 | 18 | 212 | 4 | Germ cell neoplasia in situ |
| 7 | 103635491 | G | A | . | Substitution | RELN | c.2399C>T_p.Ser800Phe | 0 | 123 | 15 | 127 | 4 | Germ cell neoplasia in situ |
| 11 | 134178690 | T | TG | . | Insertion | NCAPD3 | c.2725dupC | 0 | 23 | 4 | 21 | 4 | Germ cell neoplasia in situ |
| 1 | 120890143 | A | G | . | Substitution | PPIAL4B | c.323A>G_p.Asn108Ser | 0 | 25 | 10 | 27 | 4 | Embryonal carcinoma |
| 1 | 155942007 | G | C | . | Substitution | RXFP4 | c.298G>C_p.Glu100Gln | 0 | 80 | 29 | 92 | 4 | Embryonal carcinoma |
| 1 | 210684141 | GGA | G | rs144706702 | Deletion | KCNH1 | c.2113-5_2113-4delTC | 0 | 23 | 6 | 50 | 4 | Embryonal carcinoma |
| 1 | 248388532 | T | TCACTTTTTCTGTGAGGCACCCACCATGCTGAGGCTGGCCTG | . | Insertion | OR2T6 | c.924_925insCACTTTTTCTGTGAGGCACCCACCATGCTGAGGCTGGCCTG | 0 | 109 | 23 | 178 | 4 | Embryonal carcinoma |
| 2 | 164915923 | G | A | . | Substitution | SLC38A11 | c.500C>T_p.Ala167Val | 0 | 240 | 45 | 236 | 4 | Embryonal carcinoma |
| 3 | 53104053 | C | CCAACAGCAAGGAGAAG | . | Insertion | RFT1 | c.1001_1002insCTTCTCCTTGCTGTTG | 0 | 119 | 9 | 92 | 4 | Embryonal carcinoma |
| 3 | 108501781 | C | A | . | Substitution | MYH15 | c.330G>T_p.Met110Ile | 0 | 143 | 33 | 87 | 4 | Embryonal carcinoma |
| 5 | 74722480 | C | T | . | Substitution | GFM2 | c.2110G>A_p.Val704Ile | 0 | 163 | 60 | 221 | 4 | Embryonal carcinoma |
| 7 | 103635491 | G | A | . | Substitution | RELN | c.2399C>T_p.Ser800Phe | 0 | 123 | 38 | 176 | 4 | Embryonal carcinoma |
| 7 | 108225683 | G | C | . | Substitution | NRCAM | c.722C>G_p.Thr241Ser | 0 | 53 | 8 | 74 | 4 | Embryonal carcinoma |
| 7 | 128815812 | G | A | . | Substitution | CCDC136 | c.3244G>A_p.Glu1082Lys | 0 | 74 | 33 | 148 | 4 | Embryonal carcinoma |
| 10 | 12179100 | T | TACTCTGAGCAGATGAATCAGGTTCTGAAGGCATCATCCAGTCG | . | Insertion | NUDT5 | c.163_164insCGACTGGATGATGCCTTCAGAACCTGATTCATCTGCTCAGAGT | 0 | 113 | 18 | 82 | 4 | Embryonal carcinoma |
| 10 | 79305162 | A | G | . | Substitution | ZMIZ1 | c.1999-2A>G | 0 | 106 | 38 | 82 | 4 | Embryonal carcinoma |
| 10 | 124942972 | A | G | . | Substitution | ZRANB1 | c.479A>G_p.Tyr160Cys | 0 | 154 | 53 | 125 | 4 | Embryonal carcinoma |

**Table S4. Summary of insertion/deletions and single nucleotide variants**

| Chromosome | Position | Reference allele | Alternate allele | dbSNP | Type of mutation | Gene | Mutation | Normal alternate allele count | Normal total allele count | Tumor alternate allele count | Tumor total allele count | Patient | Histological type |
|---|---|---|---|---|---|---|---|---|---|---|---|---|---|
| 11 | 66699589 | G | GATGGAGTCTGCCTTTTCCCGAATCTTGTCGGCGTGGA | . | Insertion | SPTBN2 | c.3592_3593insTCCACGCCGACAAGATTCGGGAAAAGGCAGACTCCAT | 0 | 39 | 31 | 68 | 4 | Embryonal carcinoma |
| 11 | 116858294 | CTGT | C | rs537893827 | Deletion | SIK3 | c.2844_2846delACA_p.Gln953del | 0 | 49 | 4 | 37 | 4 | Embryonal carcinoma |
| 14 | 39399753 | A | ATAACCACAGGAATTCTACATTAAAAAAAAGCTAAATGTCTTC | . | Insertion | FBXO33 | c.1430_1431insGAAGACATTTAGCTTTTTTTTAATGTAGAATTCCTGTGGTTA_p.Ile477delinsMetLysThrPheSerPhePheLeuMetX | 0 | 174 | 37 | 173 | 4 | Embryonal carcinoma |
| 15 | 73551098 | C | CCAGG | . | Insertion | REC114 | c.494_495insCAGG | 0 | 51 | 13 | 43 | 4 | Embryonal carcinoma |
| 17 | 4797720 | C | CA | . | Insertion | PSMB6 | c.341_342insA | 0 | 30 | 12 | 35 | 4 | Embryonal carcinoma |
| 19 | 6697477 | A | G | . | Substitution | C3 | c.2663T>C_p.Ile888Thr | 0 | 34 | 5 | 29 | 4 | Embryonal carcinoma |
| 19 | 8888826 | T | TCCGA | . | Insertion | MUC16 | c.40672_40673insTCGG | 0 | 38 | 5 | 27 | 4 | Embryonal carcinoma |
| 19 | 52842152 | C | CGCCAGATATGAATTATATGCGAAAGCCTCATCACAAACCT | . | Insertion | ZNF468 | c.143-2_143-1insAGGTTTGTGATGAGGCTTTCGCATATAATTCATATCTGGC | 0 | 145 | 11 | 121 | 4 | Embryonal carcinoma |
| 20 | 45841257 | A | AC | . | Insertion | SNX21 | c.1044dupC | 0 | 52 | 7 | 46 | 4 | Embryonal carcinoma |
| 1 | 44804642 | C | T | . | Substitution | PLK3 | c.1506-8C>T | 0 | 44 | 7 | 48 | 5 | Germ cell neoplasia in situ |
| 6 | 43042832 | C | G | . | Substitution | CUL7 | c.3867G>C_p.Leu1289Phe | 0 | 51 | 5 | 23 | 5 | Germ cell neoplasia in situ |
| 7 | 103366131 | T | G | . | Substitution | PSMC2 | c.812T>G_p.Leu271Arg | 0 | 38 | 11 | 72 | 5 | Germ cell neoplasia in situ |
| 8 | 30848456 | C | A | . | Substitution | TEX15 | c.1402G>T_p.Ala468Ser | 0 | 133 | 17 | 149 | 5 | Germ cell neoplasia in situ |
| 9 | 34343424 | G | A | . | Substitution | NUDT2 | c.428G>A_p.Cys143Tyr | 0 | 88 | 9 | 65 | 5 | Germ cell neoplasia in situ |
| 10 | 49516386 | A | T | . | Substitution | ERCC6-PGBD3 | c.2133T>A_p.Phe711Leu | 0 | 79 | 9 | 87 | 5 | Germ cell neoplasia in situ |
| 17 | 81645290 | T | TG | . | Insertion | TSPAN10 | c.342dupG | 0 | 31 | 4 | 20 | 5 | Germ cell neoplasia in situ |
| 19 | 8900303 | G | GTT | . | Insertion | MUC16 | c.38938_38939insAA | 0 | 28 | 3 | 20 | 5 | Germ cell neoplasia in situ |
| 19 | 8900305 | GGC | G | . | Deletion | MUC16 | c.38935_38936delGC | 0 | 30 | 3 | 20 | 5 | Germ cell neoplasia in situ |

**Table S4. Summary of insertion/deletions and single nucleotide variants**

| Chromosome | Position | Reference allele | Alternate allele | dbSNP | Type of mutation | Gene | Mutation | Normal alternate allele count | Normal total allele count | Tumor alternate allele count | Tumor total allele count | Patient | Histological type |
|---|---|---|---|---|---|---|---|---|---|---|---|---|---|
| 20 | 64074299 | C | G | . | Substitution | RGS19 | c.307G>C_p.Val103Leu | 0 | 46 | 6 | 61 | 5 | Germ cell neoplasia in situ |
| 23 | 40692880 | G | T | . | Substitution | MED14 | c.1673C>A_p.Pro558His | 0 | 66 | 11 | 59 | 5 | Germ cell neoplasia in situ |
| 1 | 17607907 | A | T | . | Substitution | ARHGEF10L | c.539A>T_p.Asp180Val | 0 | 88 | 17 | 68 | 5 | Embryonal carcinoma |
| 1 | 66970957 | C | T | . | Substitution | MIER1 | c.763C>T_p.Gln255X | 0 | 88 | 21 | 80 | 5 | Embryonal carcinoma |
| 1 | 77929344 | C | T | . | Substitution | NEXN | c.701C>T_p.Thr234Ile | 0 | 55 | 10 | 37 | 5 | Embryonal carcinoma |
| 1 | 247100744 | G | GTGCGAT | . | Insertion | ZNF669 | c.1024_1025insATCGCA_ p.Ala342delinsAspArgThr | 0 | 31 | 6 | 47 | 5 | Embryonal carcinoma |
| 2 | 160278647 | T | G | . | Substitution | RBMS1 | c.855A>C_p.Leu285Phe | 0 | 83 | 40 | 103 | 5 | Embryonal carcinoma |
| 3 | 77493340 | C | A | . | Substitution | ROBO2 | c.764C>A_p.Thr255Asn | 0 | 116 | 58 | 186 | 5 | Embryonal carcinoma |
| 4 | 40354234 | C | CATGATGT GGGTGAA AGCTGCC T | . | Insertion | CHRNA9 | c.1154_1155insATGATGT GGGTGAAAGCTGCCT | 0 | 77 | 14 | 58 | 5 | Embryonal carcinoma |
| 4 | 40354236 | A | AGCCCGC ACCACAG TAGAGAG CG | . | Insertion | CHRNA9 | c.1157_1158insCCCGCAC CACAGTAGAGAGCGG | 0 | 77 | 7 | 51 | 5 | Embryonal carcinoma |
| 4 | 48543932 | GAGA | G | . | Deletion | FRYL | c.5464_5466delTCT_ p.Ser1822del | 0 | 97 | 24 | 103 | 5 | Embryonal carcinoma |
| 5 | 179801903 | T | C | rs199501404 | Substitution | MGAT4B | c.164A>G_p.Gln55Arg | 0 | 52 | 27 | 79 | 5 | Embryonal carcinoma |
| 6 | 31637500 | C | T | . | Substitution | PRRC2A | c.6388C>T_p.Pro2130Ser | 0 | 90 | 34 | 127 | 5 | Embryonal carcinoma |
| 6 | 43042832 | C | G | . | Substitution | CUL7 | c.3867G>C_p.Leu1289Phe | 0 | 51 | 5 | 21 | 5 | Embryonal carcinoma |
| 6 | 63684452 | G | C | . | Substitution | PHF3 | c.730G>C_p.Gly244Arg | 0 | 26 | 6 | 23 | 5 | Embryonal carcinoma |
| 7 | 92581531 | A | G | . | Substitution | FAM133B | c.67T>C_p.Tyr23His | 0 | 95 | 65 | 192 | 5 | Embryonal carcinoma |
| 7 | 103366131 | T | G | . | Substitution | PSMC2 | c.812T>G_p.Leu271Arg | 0 | 38 | 11 | 57 | 5 | Embryonal carcinoma |
| 8 | 30848456 | C | A | . | Substitution | TEX15 | c.1402G>T_p.Ala468Ser | 0 | 132 | 53 | 223 | 5 | Embryonal carcinoma |
| 8 | 84862334 | G | A | . | Substitution | RALYL | c.452G>A_p.Arg151His | 0 | 28 | 23 | 79 | 5 | Embryonal carcinoma |
| 8 | 94646192 | T | C | . | Substitution | ESRP1 | c.400T>C_p.Tyr134His | 0 | 228 | 43 | 225 | 5 | Embryonal carcinoma |
| 9 | 34343424 | G | A | . | Substitution | NUDT2 | c.428G>A_p.Cys143Tyr | 0 | 88 | 12 | 52 | 5 | Embryonal carcinoma |
| 10 | 49516386 | A | T | . | Substitution | ERCC6-PGBD3 | c.2133T>A_p.Phe711Leu | 0 | 79 | 23 | 67 | 5 | Embryonal carcinoma |
| 11 | 2570703 | G | A | . | Substitution | KCNQ1 | c.553G>A_p.Val185Met | 0 | 46 | 14 | 40 | 5 | Embryonal carcinoma |
| 19 | 23135966 | C | A | . | Substitution | ZNF730 | c.149C>A_p.Pro50Gln | 0 | 89 | 13 | 51 | 5 | Embryonal carcinoma |

**Table S4. Summary of insertion/deletions and single nucleotide variants**

| Chromosome | Position | Reference allele | Alternate allele | dbSNP | Type of mutation | Gene | Mutation | Normal alternate allele count | Normal total allele count | Tumor alternate allele count | Tumor total allele count | Patient | Histological type |
|---|---|---|---|---|---|---|---|---|---|---|---|---|---|
| 19 | 51125018 | CGGAAGGACAGACAAGTAAACTGCTGACGATGCAGAGTTCCGTGACGGTGCA | C | . | Deletion | SIGLEC9 | c.51_101del51_p.Gln18_Gly34del | 0 | 163 | 12 | 68 | 5 | Embryonal carcinoma |
| 20 | 20277258 | G | C | rs41310163 | Substitution | CFAP61 | c.2596G>C_p.Val866Leu | 0 | 33 | 21 | 56 | 5 | Embryonal carcinoma |
| 20 | 64074299 | C | G | . | Substitution | RGS19 | c.307G>C_p.Val103Leu | 0 | 46 | 24 | 57 | 5 | Embryonal carcinoma |
| 21 | 18365189 | G | GCTTCAT | . | Insertion | TMPRSS15 | c.723_724insATGAAG_p.Thr241_His242insMetLys | 0 | 149 | 51 | 312 | 5 | Embryonal carcinoma |
| 23 | 40692880 | G | T | . | Substitution | MED14 | c.1673C>A_p.Pro558His | 0 | 63 | 17 | 48 | 5 | Embryonal carcinoma |
| 23 | 133324275 | G | A | . | Substitution | GPC4 | c.581C>T_p.Thr194Met | 0 | 80 | 56 | 104 | 5 | Embryonal carcinoma |
